# A new family of urea-based low molecular-weight organogelators for environmental remediation: the influence of structure†
†Electronic supplementary information (ESI) available: Materials synthesis and characterisation, single crystal X-ray crystallography and rheology. CCDC 1846049–1846051. For ESI and crystallographic data in CIF or other electronic format see DOI: 10.1039/c8sm01682h


**DOI:** 10.1039/c8sm01682h

**Published:** 2018-10-11

**Authors:** William J. Peveler, Hollie Packman, Shirin Alexander, Raamanand R. Chauhan, Lilian M. Hayes, Thomas J. Macdonald, Jeremy K. Cockcroft, Sarah Rogers, Dirk G. A. L. Aarts, Claire J. Carmalt, Ivan P. Parkin, Joseph C. Bear

**Affiliations:** a Division of Biomedical Engineering , School of Engineering , University of Glasgow , Rankine Building , Glasgow G12 8LT , UK . Email: william.peveler@glasgow.ac.uk; b Department of Earth Science and Engineering , South Kensington Campus, Imperial College , London , SW7 2AZ , UK; c Energy Safety Research Institute (ESRI) , Swansea University , New Bay Campus , Swansea , SA1 8EN , Wales , UK; d Department of Chemistry , Physical and Theoretical Chemistry Laboratory , University of Oxford , South Parks Road , Oxford , OX1 3QZ , UK; e Department of Chemistry , University College London , 20 Gordon Street , London , WC1H 0AJ , UK; f ISIS-STFC , Rutherford Appleton Laboratory , Chilton , Oxon OX11 0QX , UK; g Department of Chemical and Pharmaceutical Sciences , Kingston University , Kingston upon Thames , Surrey , KT1 2EE , UK . Email: J.Bear@kingston.ac.uk

## Abstract

Gelation processes grant access to a wealth of soft materials with tailorable properties, in applications as diverse as environmental remediation, biomedicine and electronics. Several classes of self-assembling gelators have been studied and employ non-covalent bonds to direct assembly, but recently attention has come to focus on how the overall shape of the gelator molecule impacts its gelation. Here we study a new sub-family of low molecular weight organogelators and explore how steric rearrangement influences their gelation. The gels produced are characterised with X-ray diffraction and small-angle neutron scattering (SANS) to probe their *ex situ* and *in situ* gelation mechanisms. The best examples were then tested for environmental remediation applications, gelling petrol and oils in the presence of water and salts.

## Abstract 2

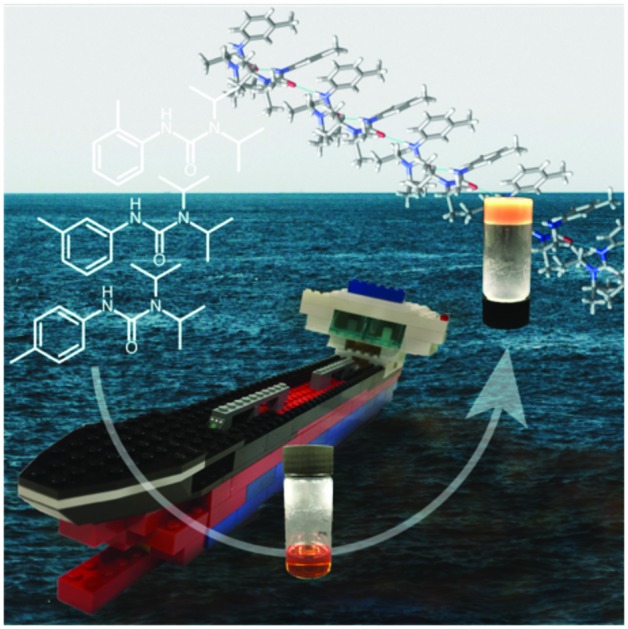
Six analogous low molecular weight organogelators are comprehensively characterised to investigate the role of small structural modifications on performance.

## Introduction

Low molecular weight organogelators (LMWOs or LMOGs) have become an important experimental material for environmental remediation of organic liquid spills, such as crude oils and refined petroleum products (engine oil, diesel *etc.*) on bodies of open water.^1^–^3^ Gelators of organic liquids allow for the solidification of these liquid spills, facilitating their physical removal using nets or meshes, and unlike absorbents, the gelator can then often be subsequently removed, to allow regeneration and purification of the spilled liquid.^4^,^5^ The gels formed can, in many cases, also selectively remove amphiphilic pollutants.^6^,^7^ Beyond this major application, organogels have also been applied as soft optoelectronic materials,^8^,^9^ stabilising matrices for sensitive materials,^10^,^11^ for cleaning and restoring artworks^12^ and in pharmaceutical products.^13^

LMWO materials operate by self-assembly into fibrillar networks (SAFINs) on cooling in an organic solvent to form a gel.^14^ This assembly is typically directed through hydrogen bonding and π–π stacking of aromatic systems to form the fibrils, and the resultant fibrils knot together, trapping the solvent in the solid-like gel structure.^15^ The shape, size and strength of the fibrillar network formed dictates the macro-scale properties of the gel, and thus its utility for practical applications.^16^ Therefore, understanding how to improve these structure–property relationships is crucial to the development of LMWOs.^17^

To create structures that undergo the favourable combination of 1D hydrogen bonding *etc.* required for fibrillar self-assembly, peptides,^18^–^20^ cholesterol derivatives^21^ and sugars, such as mannitols, have been targeted with much success.^22^,^23^ Recently, ureas have been also targeted, due to their ease and flexibility of synthesis.^24^,^25^ Ureas have been shown to provide a useful motif for controlling self-assembly and gelation, when incorporating other responsive groups into the LMWO, for example in the work of Schalley *et al.*^26^ Gross changes to the gelator structure will of course cause gel-property changes, but Gale *et al.* demonstrated a family of aromatic ureas, and explored the more subtle influence of a range of electron donating or withdrawing aryl substituents on gelation.^27^

**Fig. 1:**
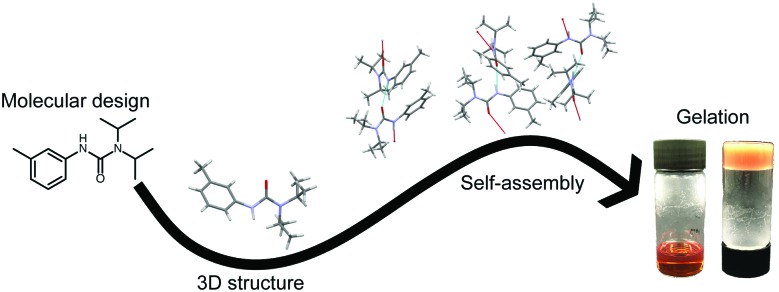
Schematic of urea gelation, directed by 1D H-bonding to form long chains, which then self-assemble into the overall fibrillary network (SAFIN). The 3D shape of the LMWO strongly influences the subsequent SAFIN formation.

Ureas form their fibrillar structure though uni-directional hydrogen bonding between the R–NH moieties of one urea, and the carbonyl oxygen of the next (Fig. 1).^28^ By controlling the number, sterics and electron density of the RNH segments, the gelation properties can be modified. Gale *et al.* showed that small changes in sterics and substituent effects can cause large changes in structure. This, in turn radically changes the gelation power of the materials, facilitating tuning of the gelation properties.^27^ Critically, the synthesis of ureas from industrially available, low cost isocyanates (precursors for the manufacture of polyurethanes) and simple amines is facile and high yielding at room temperature, making industrial scale production of such materials more achievable. This is coupled with recent results demonstrating the utility of urea gelators for the gelation of oil on water.^29^

Herein we describe a new sub-family of urea LMWOs discovered during a larger screening exercise on urea and carbamate LMWOs. We demonstrate the potential for environmental remediation with these materials, and exploit the homologous nature of this family of gelators to further explore the relationship between structure and gelation.^30^ We have performed microscopy, crystallography and neutron scattering to understand the self-assembly properties of the gels and derived xerogels (with excess solvent removed) and from this show how very small changes to LMWO structure have big effects on the gels produced.

## Results and discussion

**Scheme 1:**
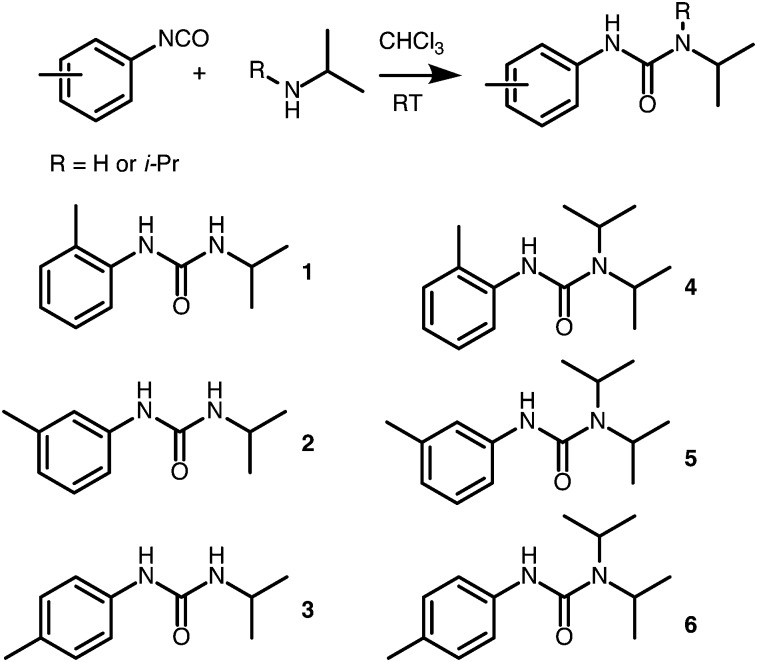
Structure of the gelators used in this study along with a general synthetic scheme from isocyanate precursors.

Six LMWOs were synthesised (Scheme 1) as part of a larger test set and assessed for their gelation of 5 solvents of varying polarity. The compounds were characterised by NMR and mass spectrometry, as well as IR and melting point analysis. Full details and figures are given in the ESI.† Molecules **1**, **3** and **6** appear to have been previously synthesized as reaction intermediates but not tested as LMWOs;^31^,^32^ and **2**, **4** and **5** were to the best of our knowledge, novel compounds.

LMWOs **1**, **2**, and **3**, did not form stable gels with most or all of the solvents tested, whereas **4**, **5**, and **6** all formed gels at 10% w/v in non-polar solvents (cyclohexane, petroleum ether (PE) and 1-octadecene (ODE)). At lower concentrations gelation was maintained for **5** and **6** but not **4**, particularly in petroleum ether and ODE. The critical gelation concentration (CGC) for each was determined to be lowest for ODE gels (5%, 0.6% and 0.2% w/v for **4**, **5**, and **6** respectively – Table 1).

**Table 1:** The gelation properties of the 6 gelators in solvents of varying polarity. G indicates a gel formed and the CGC estimated from inversion testing is given in brackets (units% w/v). X indicates the material was insoluble and D indicated that the LMWO dissolved but did not gel the solvent at 10% w/v

LMWO	DMSO	Chloroform	Petroleum ether (60–80)	Cyclohexane	ODE
**1**	D	D	X	X	G (1.3)
**2**	D	D	X	X	X
**3**	D	D	X	X	G (2.5)
**4**	D	D	G (10.0)	G (5.0)	G (5.0)
**5**	D	D	G (0.5)	G (5.0)	G (0.6)
**6**	D	D	G (0.3)	G (1.7)	G (0.2)

**Fig. 2:**
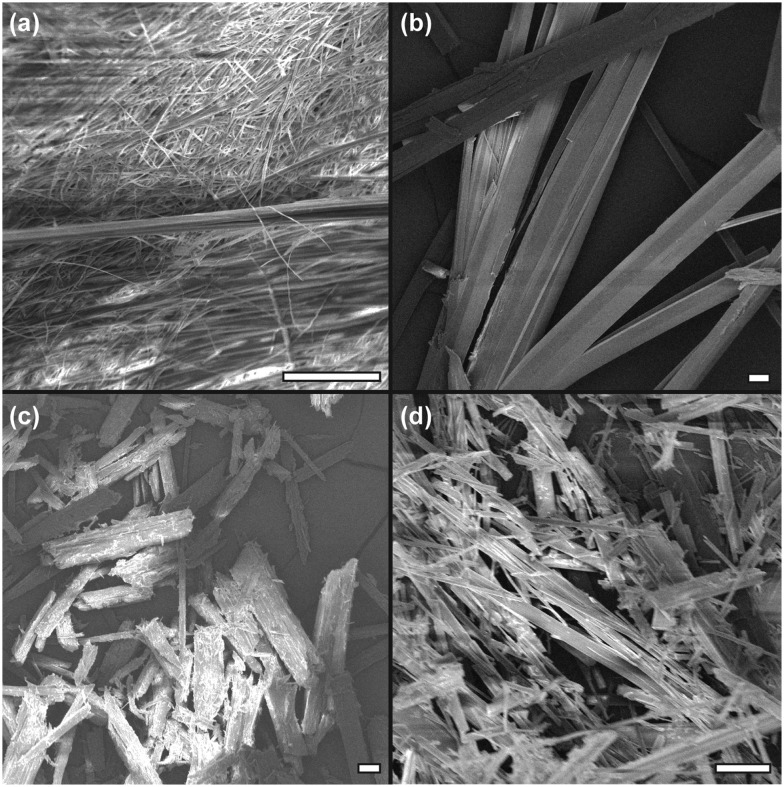
SEM images of xerogels from various solvents at 5% w/v. (a) **5** from PE, (b) **4** from cyclohexane, (c) **5** from cyclohexane and (d) **6** from cyclohexane. All scale bars 20 μm.

The fibrillar network of the gels produced in cyclohexane was a large, almost crystalline network confirmed by SEM micrographs of the xerogels (dried under vacuum at RT for 12 hours), with straight, rigid fibrils/crystallites observed (Fig. 2). These were approximately 40 μm in diameter for **4**, ∼30 μm for **5** and less than 10 μm for **6**. In petroleum ether, **5** showed a much more interwoven network of fine fibres (diameter of ∼1 μm), and this more fibrous structure corresponds with the improved CGC in the solvent in comparison to cyclohexane. It was not possible to obtain SEM of the ODE gels due to the difficulty in removing the solvent whilst maintaining the network structure, but based on visual observation and CGC properties, it is hypothesised that they would resemble the finer fibrils shown in Fig. 2(a). Additional (higher-magnification) SEM images are given in Fig. S7 (ESI†). The materials were tested for thixotropic properties, but little evidence was found for this type of behaviour. However vigorous centrifugation allowed recovery of the gelator from the organic phase, suggesting the materials and the organic liquids gelled might be recyclable if used in environmental remediation (Fig. S8, ESI†).

### Single crystal X-ray diffraction

The almost crystalline nature of the gels led to attempts at growing single crystals of **4**, **5**, and **6** for single-crystal X-ray diffraction (SCXRD), which proved to be exacting. Although it is understood that these crystals may not be fully representative of the packing inside the SAFINs of the gel, they do at least give some idea of intermolecular connectivity and packing occurring.^17^ Of particular interest was the physical effect of the tolyl substituent position on self-assembly, as the gels demonstrated very different properties depending on this moiety. It was also of interest to observe the nature of the single hydrogen bond, given that the equivalent molecules with a double H-bond potential (**1**, **2** and **3**) did not form gels reliably.

**Fig. 3:**
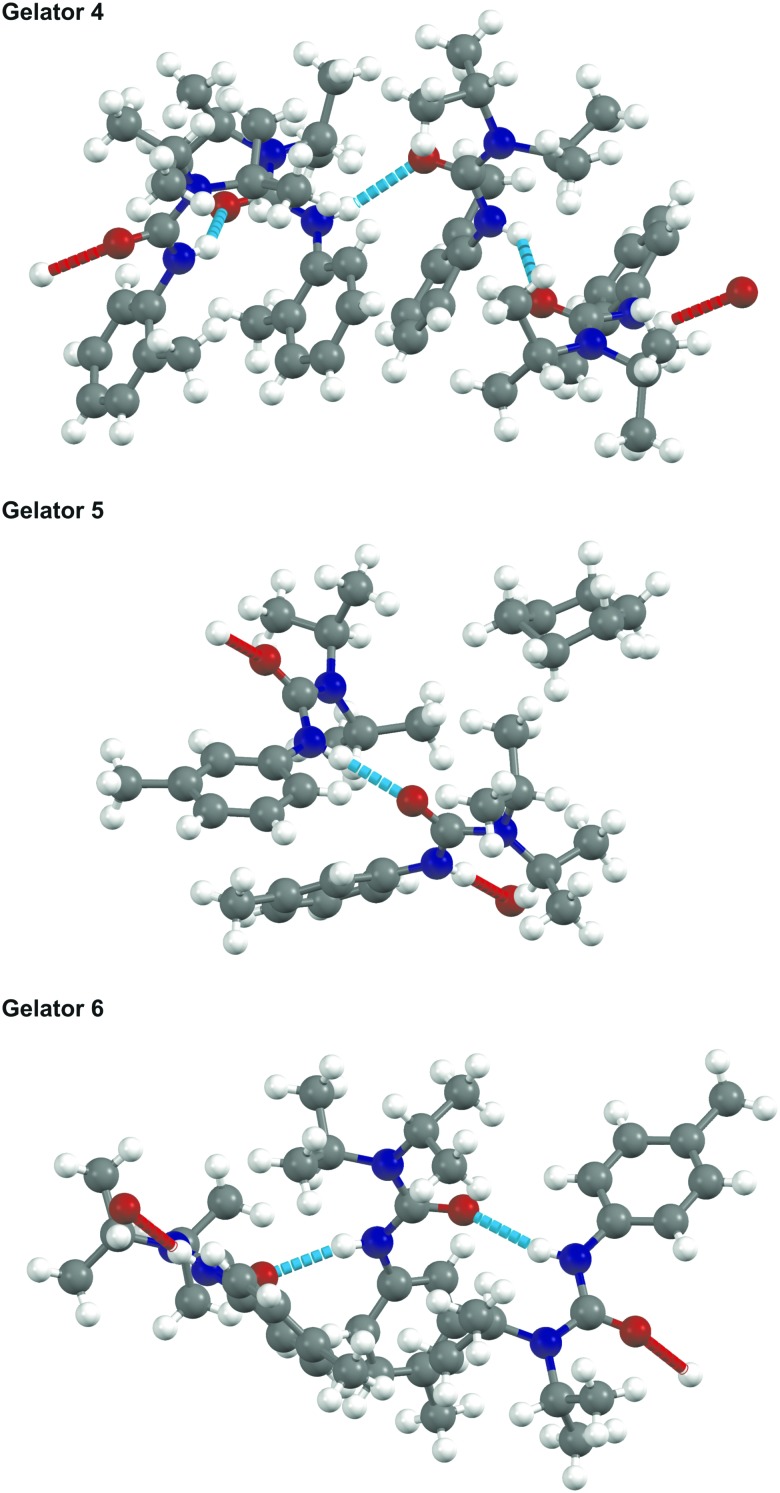
SCXRD structures of gelators **4**, **5** and **6**, showing the number of independent molecules in the asymmetric unit cell of the crystallographic cell (4, 2, and 3, respectively) and the chain network formed by N–H···O hydrogen-bonding.

The crystal structure of gelators **4**, **5**, and **6** were determined and full crystallographic details are available in the ESI.† None of these gelators formed simple crystal structures: all three resulted in structures with more than one crystallographic molecule per unit cell (*i.e. Z*′ is >1) as shown in Fig. 3. All three form 1-D hydrogen bonded networks with weak van der Waals interactions in the other directions, consistent with their properties as fibre-forming gelators. The diisopropylamine group prevents effective π–π interactions between the aromatic rings and deters an easy route to a crystalline solid.

The crystal structure of **6** is densely packed and contains three hydrogen-bonded gelator molecules per asymmetric unit, without solvent incorporation. By contrast, **5** crystallises with two gelator molecules in the asymmetric unit and with voids in the structure filled by the cyclohexane solvent used in crystallisation.

The determination of the crystal structure of gelator **4** proved problematic as the molecules could not pack efficiently and form a 1-D hydrogen-bonded chain simultaneously. Consequently, there is a wave-like nature to the orientation of adjacent aromatic rings resulting in an incommensurate structure. An approximate commensurate structure could be derived from the data with *Z*′ = 4 as shown. A full explanation and demonstration of the incommensurate nature of the data is shown in Fig. S1, ESI.†

Although there is little correlation between predicting the effectiveness of the gelator and the nature of the crystal structure, we can say that a large amount of disorder or high *Z*′ seems to be an indicator for a compound to be an LMWO. However, gelator **4** has the highest *Z*′ but has higher CGC values for gelling petroleum ether, cyclohexane, and 1-octadecene than gelators **5** and **6** indicating that other factors, such as interactions between gelator and solvent, need to be considered.

### Small-angle neutron scattering

To accompany the *ex situ* SEM and SCXRD analysis, *in situ* small-angle neutron scattering (SANS) data were collected for the cyclohexane gels in order to determine their structural characteristics at various concentrations and temperature. Due to the large size of the fibres and the *Q* range studied here, it was not possible to fit the SANS patterns to a specific shape. However, the data were fitted using shape independent models such as power law and fractal. With these models, it can be confirmed that we are observing a gel network or surface of a large fibrillar structure. Cyclohexane was used as the model solvent as it was hard to assess the PE and ODE gels, due to the complex mixed nature of the former solvent and the high boiling point and difficulty in obtaining d-versions of the latter.

**Fig. 4:**
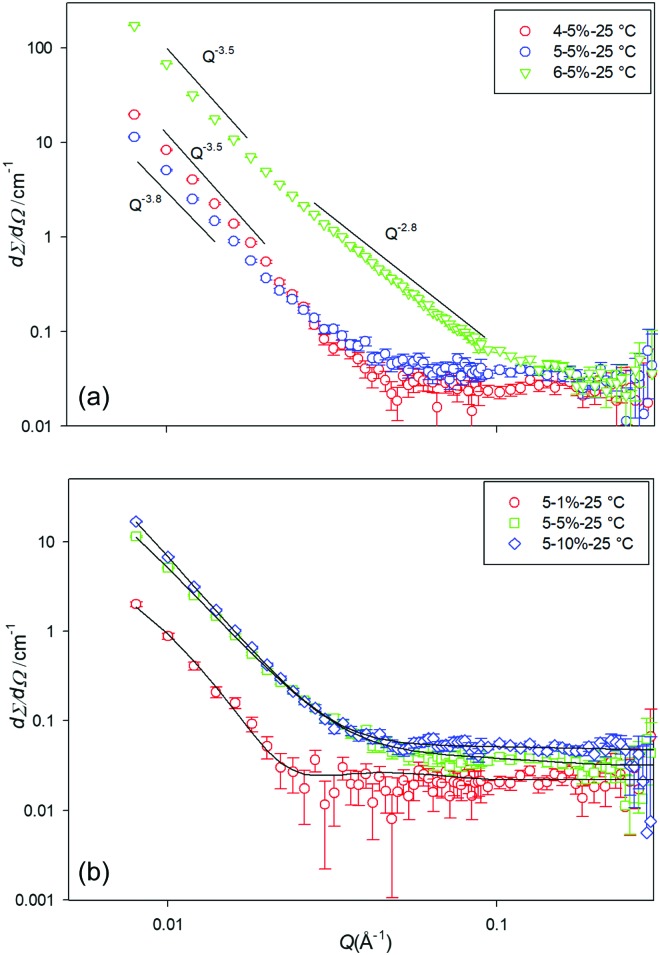
SANS profiles for (a) the three different gelators at 5% w/v, and (b) gelator **5** at a range of concentrations, at 25 °C in d_12_-cylcohexane.

The scattering pattern of gelators **4**, **5** and **6** (5% w/v) at 25 °C (at which the gels are fully formed) is shown in Fig. 4a. As can be seen from the SANS profiles, gels of **4** and **5** have almost the same shape and showed the behaviour of *I*(*Q*) ∼ *Q*^–3.5^ which corresponds to the scattering from surface fractals. The scattering pattern of gels formed by **6** is slightly different. The initial slope is around –2.8 which corresponds to the scattering from gel networks or mass fractal, then the slope changes at around *Q* = 0.017 Å^–1^ to about –3.5. This corroborates with the CGC values for **4** and **5***vs.***6** in cyclohexane, where **4** and **5** have just gelled at 5% whereas **6** is already a stable at *ca.* 1.7%.

The scattering data from **4** at 1% w/v indicated unstable gels (as also evidenced by the CGC estimation) and no SANS patterns were collected. Fig. 4b, shows the scattering pattern of the gelator **5** at various concentrations. As can be seen from the scattering profile, gels are formed at various concentrations. However, no SANS pattern was observed for **5** at 1% w/v at temperatures above 25 °C, due to melting/solution of the gel network.

Data were fitted to the fractal model (the model calculates the scattering from fractal-like aggregates of spherical building blocks).^33^ The fitted data indicates that as the concertation of **5** decreases from 10% w/v to 1% w/v the radius of the particles increases from 11 Å to around 35 Å, however, the cluster correlation length decreases from around 400 Å to about 125 Å.

**Fig. 5:**
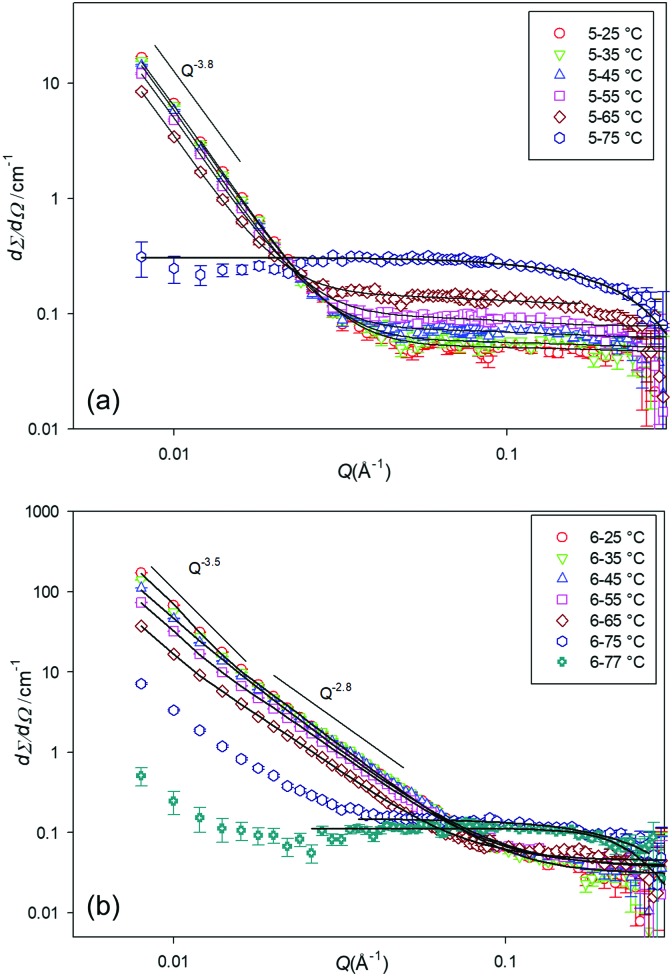
SANS profiles for (a) **5**, 10% w/v, and (b) **6**, 5% w/v, at a range of temperature, in d_12_-cylcohexane.

Fig. 5 shows the SANS profile of the **5** and **6** gels at various temperatures (25, 35, to 75 °C). As can be seen from the data the scattering intensity gradually decreases with an increase of temperature, until a certain temperature at which the gels melted and the scattering pattern changes. For gelator **5**, this temperature is distinct; at 75 °C the scattering pattern changes from cluster like objects with correlation length of around 350 Å (reflected in the SEM images) to small spherical particles with radius of around 8 Å, presumably small associated agglomerates in solution.

Gelator **6** behaves slightly differently. At 75 °C there is a mixture of spherical structures and fibres and this is expected to be the sol–gel transition temperature. At 77 °C mainly spherical particles are observed. The gels from **6** form much longer range clusters, with a correlation length of above 1000 Å and particle radius of around 5 Å at 25 °C to correlation length of around 670 Å but particle radius of around 11 Å at 65 °C, suggesting a fibrous structure. The data at 77 °C was fitted to the spherical model with radius of 11 Å.

Overall, on the basis of our *in* and *ex situ* analysis, gelator **6** seems to form the most stable gels with low CGCs and stable structures even at high temperature, as assessed by SANS. On the basis of the observed structures in SANS and under SEM, this seems to be due to a better SAFIN formation with smaller, longer fibres than **4** or **5**. More generally the restriction of the H-bonding to a single possible bond by incorporation of the second diisopropyl group leads to better gel formation. This is rationalised on the basis of an improved balance between energy of solvation and energy of crystallisation. Without this second group, the intermolecular bonding becomes strong and the molecule approaches insolubility or on crystallisation rapidly forms small precipitates rather than the desired, slow, chain/fibre formation.

### Functional testing

Materials **4**, **5** and **6** were tested for applications in environmental remediation of oil spills. We firstly tested the gelation properties of the materials in pump oil, calculating the CGC, and making rheology and calorimetric measurements. It was found that CGC was maintained at less than 1% for each of the three materials. Gels at 2% w/v were then analysed with rheometry and differential scanning calorimetry (DSC). It was found that the yield stress was in fact an order of magnitude higher for **5**, along with the greatest values of *G*′ and *G*′′ within the linear viscoelastic region. Gels of **6** also displayed solid-like behaviour, however the gels of **4** were far more liquid-like, particular at higher stresses or frequencies. (Fig. S3 and S4, ESI†).

The gelation of the material was investigated by DSC (Fig. S5 and S6, ESI†) and it was found that on cooling a solution of the gelator, **4** gelled first but with a broad transition, and the sharper transitions for **6** and **5** occurred at *ca*. 20 °C cooler with **6** having the slightly lower gelation point. On reheating, all transitions were much broader. **5** melted first at *ca*. 72 °C, followed by **6** and then **4**. These results demonstrate the significant impact of a change in substitution on the gelator. Gelator **4** forms temperature stable gels, but with little physical stability in the oil. Gelators **5** and **6** form structures with lower *T*_gel_, but these manifest as physically more tough gels. The difference between ordering of melt and gelation temperatures in each direction is likely a manifestation in the kinetics of gelation, with the onset of crystallisation for **6** occurring a little before **5**, but taking longer to complete (Fig. S5, ESI†). It is also of note that the thermal profile of these gels is very different in the pump oil, in comparison to the d-cyclohexane used for the SANS measurements. The evidence of trends with gelator structure remain, but this again highlights the importance of solvent on gelation.

**Fig. 6:**
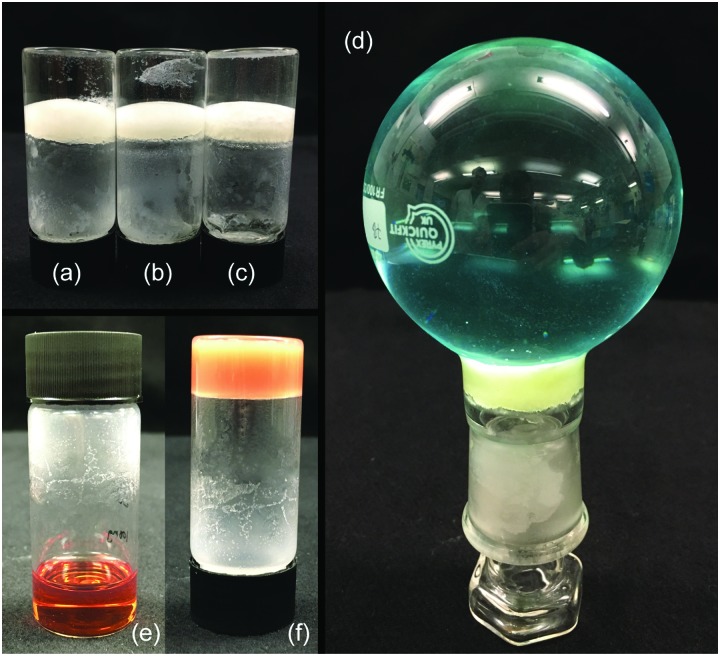
Images of gels of **5** and **6** in various polluting liquids formed on the surface of both DI and sea water: 5% pump oil gels of (a) **6** with water, (b) **5** with water and (c) **5** with sea water, each supporting 5 mL of water. (d) 120 g of water (coloured with CuSO_4_) supported by a 5% gel of **6** and (e) sol–(f) gel phases of **5** in dirty engine oil.

As a final practical and qualitative test, we explored whether the gelator was capable of gelling certain relevant liquids in the presence of water, which might interfere with the hydrogen bonding network of the gel, and in particular sea water, where salts may also hinder gelation.^34^ Although cyclohexane is a reasonable mimic for some organic liquid spills, the results with ODE, petroleum ether and pump oil demonstrate the importance of using a variety of solvents. We also investigated a dirty engine oil as a realistic mimic for the aged oils spilled in a marine collision. Gelation was attempted on either DI or sea water. We demonstrated that LMWO **5** and **6** could gel on the surface of the water forming a solid plug that would retain a weight of water above – Fig. 6.

Overall, it appears that the more extended LMWO shapes with *meta*- and *para*-substitution on the aryl group are able to form more stable gel structures, due to changes in their molecular packing. Whilst only two of the LMWOs discussed here can claim to be “super-gelators” in certain solvents, of particular interest is the finding that bulky *N*-substitution on the urea group actually improves gelation properties, by rendering the LMWO more immediately soluble in the organic liquids of interest, and this may lead to gelators that are easier to apply *in situ* at oil spills. It is notable that salts did not adversely influence the formation of the gels on an aqueous interface. Finally, this study does highlight the need for careful LMWO selection based on the liquid that need to be gelled, and further studies will explore modification of LMWO electron density as well as sterics, to optimise material properties.

## Conclusions

We have demonstrated a new family of self-assembling LMWOs, based on a subset of a large group of urea-based molecules. We have shown that 2 members of this family (novel compound **5** and compound **6**) are good gelators for a range of environmentally polluting liquids, such as petrol and oils, and can form stable gels at less than 1% w/v in certain cases. We have explored how a small modification in gelator structure has a large impact on the self-assembly properties of the material, using both *in situ* (SANS/rheology) and *ex situ* (SCXRD/SEM) analysis. In particular, the restriction of available hydrogen bonding sites and an increase in steric bulk actually improved gelation properties, through improved solubility in relevant solvents. Further to this, modification of the symmetry, and hence sterics of the aryl group dramatically changed the self-assembly thermodynamics and kinetics, and corresponding gel strengths.

Whilst this family of gelators may not be as strong as existing molecules such as mannitols or cholesterols, they are facile to synthesise in bulk quantities, from industrial precursors, and are amenable to a range of structural modifications. It is hoped that through a better understanding of the structure–property relationships of this family of molecules, a useful new class of materials can be developed in future.

## Materials and methods

A large-scale screening exercise was undertaken to discover useful urea and carbamate gelators that would gel petrol, solvents and viscous engine oils in the absence and presence of water. From this larger compound set, a promising class of gelalators based on ureas formed between tolyl-isocyanate and isopropylamines were focussed on. In particular, it was found that the ureas formed with diisopropylamine were good target LMWOs.

In a typical synthesis, tolyl-isocyanate (4.0 g, 30 mmol), was added dropwise to a vigorously stirred solution of the relevant isopropyl amine (30 mmol) in 30 mL chloroform. A lid was placed loosely on the flask to prevent excessive evaporation of the solvent, and the reaction stirred overnight (18 h) at room temperature. The reactions produced either a clear yellow solution or precipitated a white/off-white solid. The solutions were dried under reduced pressure on a rotary evaporator, to cause precipitation. All solid products were then triturated with ice-cold hexane. Full methods and characterisation are given in the ESI.†

The materials produced were tested for gelation at 10% w/v in chloroform, dimethylsulfoxide (DMSO), petroleum ether 60–80 °C, cyclohexane and 1-octadecene (ODE), to attempt gelation at a range of solvent polarities. The powdered LMWO was weighed (100 mg) into a glass tube and 1 mL of solvent was added, before capping the tube and heating to below the boiling point of the solvent. The LMWO either dissolved or remained insoluble. If a solution formed, then on cooling the tube was inverted to assess whether a gel had formed. If gelation was successful then the test was repeated in that solvent at lower concentrations. Successful gelators were also trialled against a viscous engine oil and pump oil in the presence and absence of water. Additional methods are given in the ESI.†

The SANS measurements were carried out on the successful gelators (at various concentrations in d_12_-cyclohexane) at the ISIS facility, Rutherford Appleton Laboratory, UK on the LOQ instrument. LOQ is a fixed-sample detector instrument that uses a white neutron beam with wavelengths between 2.2 and 10.0 Å to provide a *Q*-range of 0.006–1.0 Å^–1^. All samples were measured in 2 mm path-length rectangular quartz cells, and raw SANS data were normalized by subtracting the scattering of the empty cell and a solvent background. Variable temperature was used to gain information on gel formation and melting/dissolution. The Mantid^35^ programs was used for data reduction and SANS data were fitted using the fitting program SansView^36^ which uses an iterative, least-squares fitting process.

Crystal structures are accessible from the CCDC with deposit numbers: **4**: CCDC ; 1846049, **5**: CCDC ; 1846050 and **6**: CCDC ; 1846051. Summary data are provided in ESI,† Tables S1–S3.

## Conflicts of interest

There are no conflicts to declare.

## Supplementary Material

Supplementary information

Crystal structure data
