# Supplementary material for: A new family of urea-based low molecular-weight organogelators for environmental remediation: the influence of structure
Source: Soft Matter. 2018 Oct 11;14(43):8821–7. doi: 10.1039/c8sm01682h (PMC6256360; doi:10.1039/c8sm01682h)
Supplement: Supplementary file 1 [file SM-014-C8SM01682H-s001.pdf]

Electronic Supplementary Material (ESI) for Soft Matter.
This journal is © The Royal Society of Chemistry 2018

Supporting Information

A new family of urea-based low molecular-weight organogelators for environmental remediation: the influence of structure

William J Peveler[a]*, Hollie Packman[b], Shirin Alexander[c], Raamanand R Chauhan[d], Lilian M Hayes[e], Thomas J Macdonald[e], Jeremy K Cockcroft[e], Sarah Rogers[f], Dirk G A L Aarts[d], Claire J Carmalt[e], Ivan P Parkin[e] and Joseph C Bear[g]*

[a] Division of Biomedical Engineering, School of Engineering, University of Glasgow, Rankine Building, Glasgow G12 8LT, UK. william.peveler@glasgow.ac.uk
[b] Department of Earth Science and Engineering, South Kensington Campus, Imperial College, London, SW7 2AZ, UK
[c] Energy Safety Research Institute (ESRI), Swansea University, New Bay Campus, Swansea, SA1 8EN, Wales, UK
[d] Department of Chemistry, Physical and Theoretical Chemistry Laboratory, University of Oxford, South Parks Road, Oxford, OX1 3QZ, UK
[e] Department of Chemistry, University College London, 20 Gordon Street, London, WC1H 0AJ, UK
[f] ISIS-STFC, Rutherford Appleton Laboratory, Chilton, Oxon OX11 0QX, UK
[g] Department of Chemical and Pharmaceutical Sciences, Kingston University, Kingston upon Thames, Surrey, KT1 2EE, UK J.Bear@kingston.ac.uk

Table of Contents

Synthetic Notes 2
Synthesis of 1-6 2
NMR Spectra 4
Figure 6 Gels 7
Crystallography Data for 4, 5 and 6 8
Rheology of gels of 4, 5 and 6 at 2% w/v in pump oil 35
DSC of gels of 4, 5 and 6 at 2% w/v in pump oil 36
Additional SEM imagery 38
Gelator Recovery 39

Synthetic Notes

Reagents were purchased form Sigma Aldrich and VWR and used as shipped unless otherwise noted. All solvents were reagent grade, and DI water where used was purified to 15 MΩ cm. “Pump oil” was Leybold Spezialöl N62 mineral oil and dirty engine oil was sourced from an abandoned 2-stroke engine. Sea water was provided by Sigma Aldrich, from the Gulf of Mexico, and had been filtered before bottling. All reactions and modifications were performed at room temperature. ^{1}H and ^{13}C NMR were collected on Avance Brucker 300, 400 or 600 instruments as stated. Normal resolution mass spectrometry (MS) data were collected under positive or negative Electrospray Ionisation (ESI) on a ThermoFisher Scientific LTQ Velos or Waters LCT Premier XE and GC-MS on an ISQ single quadrupole MS Trace 1310 GC. Molecular ions or other major peaks are reported. Melting points were measured visually in glass capillaries with an Electrothermal digital melting point apparatus, with a ramp rate of 2 ^{o}C/min. SEM imagery was obtained on a Jeol 6700F FEG SEM operating at 5 kV, using gold or carbon sputter coated samples of xerogels, made in appropriate solvents and dried under high vacuum. SANS data, Rheometry, DSC and SCXRD were obtained as detailed in the appropriate sections.

Synthesis of 1-6

Synthesis of **1**: *o*-tolyl isocyanate (1.07 g, 8 mmol), was added dropwise to a vigorously stirred solution of isopropyl amine (0.47 g, 8 mmol) in 10 mL chloroform. A lid was placed loosely on the flask to prevent excessive evaporation of the solvent, and the reaction stirred overnight (18 hrs) at room temperature, producing an off-white solid. The solid product was filtered and triturated with ice-cold hexane to yield a white solid (0.81 g, 52.4%) mp 178 ^{o}C; ^{1}H NMR (400 MHz, $CDCl_3$) δ 7.38 (1H, m, *H*Ar), 7.21 (2H, m, HAr), 7.11 (1H, m, *H*Ar), 6.03 (1H, s, N*H*), 4.50 (1H, d, J = 6.8 Hz, N*H*), 4.00 (1H, sept, J = 6.6 Hz, C*H*(CH_3)$_2$), 2.27 (3H, s, ArC*H*$_3$), 1.13 (6H, d, J = 6.5 Hz, CH(C*H*$_3$)$_2$); ^{13}C NMR (125 MHz, $CDCl_3$) δ 155.7, 136.1, 133.0, 131.3, 127.3, 126.2, 125.7, 42.4, 23.4, 18.0; *m/z* (GC-MS) 193.1 [M+H]
Found in the literature[1] – compound 10a –melting point and ^{13}C NMR in agreement.

Synthesis of **2**: *m*-tolyl isocyanate (1.07 g, 8 mmol), was added dropwise to a vigorously stirred solution of isopropyl amine (0.47 g, 8 mmol) in 10 mL chloroform. A lid was placed loosely on the flask to prevent excessive evaporation of the solvent, and the reaction stirred overnight (18 hrs) at room temperature giving a white solid. The solid product was filtered and triturated with ice-cold hexane to yield a white solid (1.54 g, quantitative) mp 127 ^{o}C; ^{1}H NMR (400 MHz, $CDCl_3$) δ 7.14 (2H, m, *H*Ar), 7.06 (1H, m, *H*Ar), 6.86 (1H, m, *H*Ar), 6.75 (1H, s, N*H*), 4.97 (1H, d, J = 7.5 Hz, N*H*), 3.98 (1H, sept, J = 6.5 Hz, C*H*(CH_3)$_2$), 2.28 (3H, s, ArC*H*$_3$), 1.14 (6H, d, J = 6.4 Hz, CH(C*H*$_3$)$_2$); ^{13}C NMR (125 MHz, $CDCl_3$) δ 155.2, 139.5, 138.5, 129.2, 124.9, 122.1, 118.4, 42.4, 23.4, 21.6; *m/z* (ESI+) 193.1 [M+H]

Synthesis of **3**: *p*-tolyl isocyanate (1.07 g, 8 mmol), was added dropwise to a vigorously stirred solution of isopropyl amine (0.47 g, 8 mmol) in 10 mL chloroform. A lid was placed loosely on the flask to prevent excessive evaporation of the solvent, and the reaction stirred overnight (18 hrs) at room temperature, producing an off-white solid. The solid product was then filtered and triturated with cold acetone to yield a white solid (0.18 g, 12.5%) mp 156 ^{o}C; ^{1}H NMR (400 MHz, $CDCl_3$) δ 7.14 (2H, d, J = 8.1 Hz, $_{H}$Ar), 7.06 (2H, d, J = 8.1 Hz, *H*Ar), 6.86 (1H, s, N*H*), 5.05 (1H, m, N*H*), 3.96 (1H, sept, J = 6.6 Hz, C*H*(CH_3)$_2$), 2.28 (3H, s, ArC*H*$_3$), 1.12 (6H, d, J = 6.6 Hz, CH(C*H*3)$_2$); ^{13}C NMR (125 MHz, $CDCl_3$) δ 155.5, 135.8, 134.1, 130.1, 122.3, 42.4, 23.4, 21.0; m/z (ESI+) 193.1 [M+H]; Found in the literature[1] – compound 10b –melting point and ^{13}C NMR in agreement.

Synthesis of **4**: *o*-tolyl isocyanate (4.0 g, 30 mmol), was added dropwise to a vigorously stirred solution of diisopropyl amine (3.0 g, 30 mmol) in 30 mL chloroform. A lid was placed loosely on the flask to prevent excessive evaporation of the solvent, and the reaction stirred overnight (18 hrs) at room temperature. The reaction produced a clear yellow solution and the solution was

[1] M. Tanno, S. Sueyoshi, *Chem. Pharm. Bull.* **1987**, *35*, 1360–1371.

dried under reduced pressure on a rotary evaporator, to cause precipitation. The solid product was then triturated with acetone hexane to give a white solid (4.61 g, 65.6%) mp 140 ^{o}C; ^{1}H NMR (300 MHz, $CDCl_3$) δ 7.67 (1H, d, J = 7.9 Hz, *H*Ar), 7.09 (2H, m, *H*Ar), 6.89 (1H, m, *H*Ar), 6.03 (1H, s, N*H*), 3.96 (2H, sep, J = 6.9 Hz, C*H*(CH_3)$_2$), 2.19 (3H, s, ArC*H*$_3$), 1.27 (12H, d, J = 6.9 Hz, CH(C*H*$_3$)$_2$); ^{13}C NMR (125 MHz, $CDCl_3$) δ 154.9, 137.7, 130.4, 127.6, 126.9, 123.4, 122.3, 45.5, 21.6, 18.4. *m/z* (ES+) 235.2 [M+H]

Synthesis of **5**: *m*-tolyl isocyanate (4.0 g, 30 mmol), was added dropwise to a vigorously stirred solution of diisopropyl amine (3.0 g, 30 mmol) in 30 mL chloroform. A lid was placed loosely on the flask to prevent excessive evaporation of the solvent, and the reaction stirred overnight (18 hrs) at room temperature. The reaction produced a clear yellow solution and the solution was dried under reduced pressure on a rotary evaporator, to cause precipitation. The solid product was then triturated with acetone to give an off-white solid (5.70 g, 81.1%) mp 115 ^{o}C; ^{1}H NMR (300 MHz, $CDCl_3$) δ 7.27 (1H, m, *H*Ar), 7.15 (1H, m, *H*Ar), 7.11 (1H, m, *H*Ar), 6.81 (1H, m, *H*Ar), 6.16 (1H, s, N*H*), 3.97 (2H, m, C*H*(CH3)$_2$), 2.31 (3H, s, ArC*H*3), 1.31 (12H, d, J = 6.9 Hz, CH(C*H*$_3$)$_2$); ^{13}C NMR (125 MHz, $CDCl_3$) δ 154.8, 139.4, 138.9, 128.8, 123.6, 120.4, 116.8; 45.6, 21.7, 21.6; *m/z* (ES+) 235.2 [M+H], 469.4 [2M+H]

Synthesis of **6**: *p*-tolyl isocyanate (4.0 g, 30 mmol), was added dropwise to a vigorously stirred solution of diisopropyl amine (3.0 g, 30 mmol) in 30 mL chloroform. A lid was placed loosely on the flask to prevent excessive evaporation of the solvent, and the reaction stirred overnight (18 hrs) at room temperature. The reaction produced a clear yellow solution and the solution was dried under reduced pressure on a rotary evaporator, to cause precipitation. The solid product was then triturated with ice-cold hexane to give a white solid (6.30 g, 89.6%) mp 140 ^{o}C; ^{1}H NMR (400 MHz, $CDCl_3$) δ 7.25(2H, m, *H*Ar), 7.08 (2H, m, *H*Ar), 6.25 (1H, s, N*H*), 3.96 (2H, sept, J = 6.9 Hz, C*H*(CH_3)$_2$), 2.28 (3H, s, ArC*H*3), 0.94 (12H, d, J = 6.6 Hz, CH(C*H*$_3$)$_2$); ^{13}C NMR (125 MHz, $CDCl_3$) δ 154.9, 136.8, 132.2, 129.5, 119.9, 45.5, 21.7, 20.8; *m/z* (ES+) 235.2 [M+H], 469.3 [2M+H]. Found in the literature[2] - compound 9b – ^{13}C NMR and melting point in agreement.

[2] M. Tanno, S. Sueyoshi, S. Kamiya, *Chem. Pharm. Bull.* **1990**, *38*, 2644–2649.

NMR Spectra

All in $CDCl_3$ some traces appear, including water (δ 1.56), trace acetone (δ 2.17) and $CHCl_3$ (δ 7.26).

Molecule 1

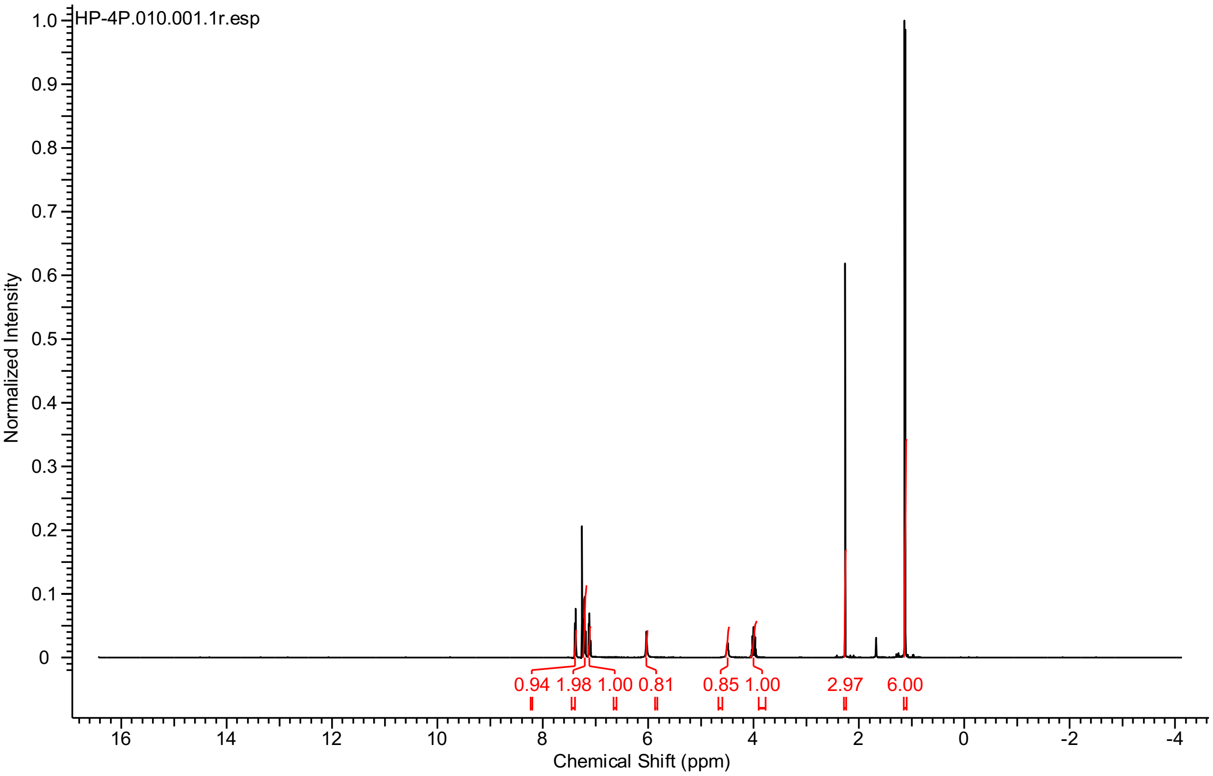

Molecule 2

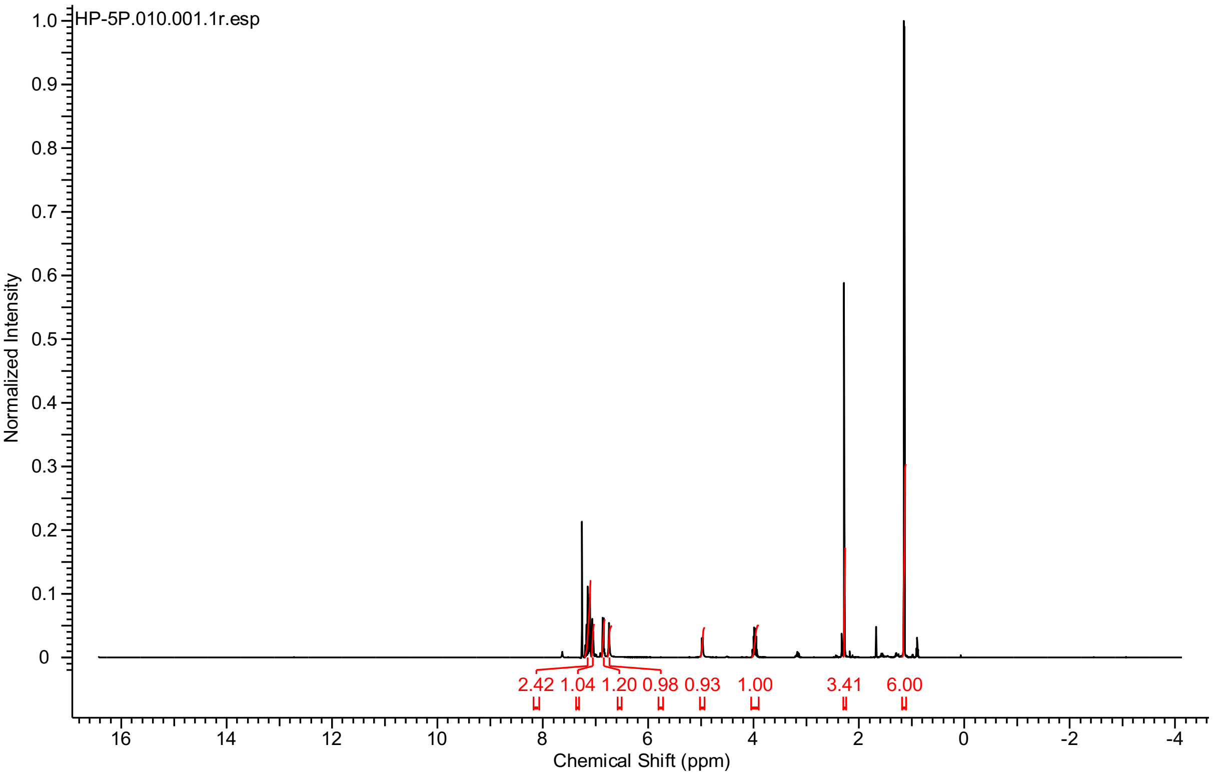

Molecule 3

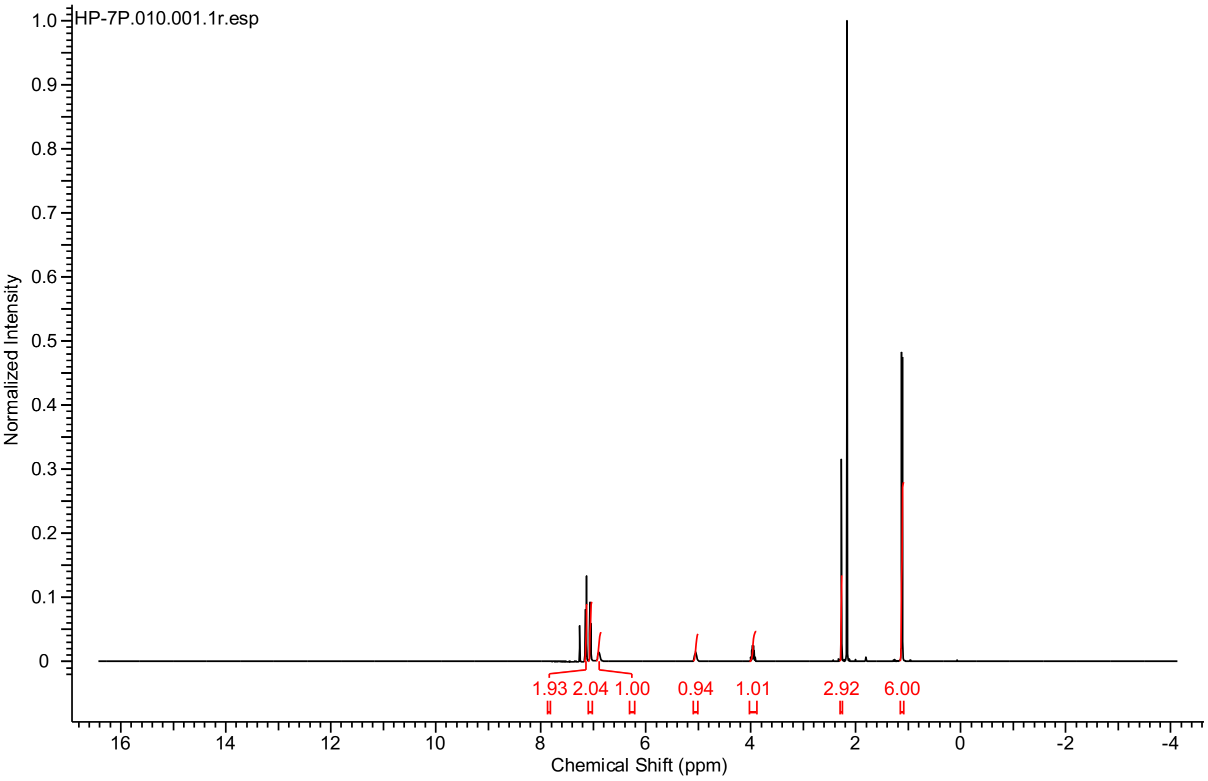

Molecule 4

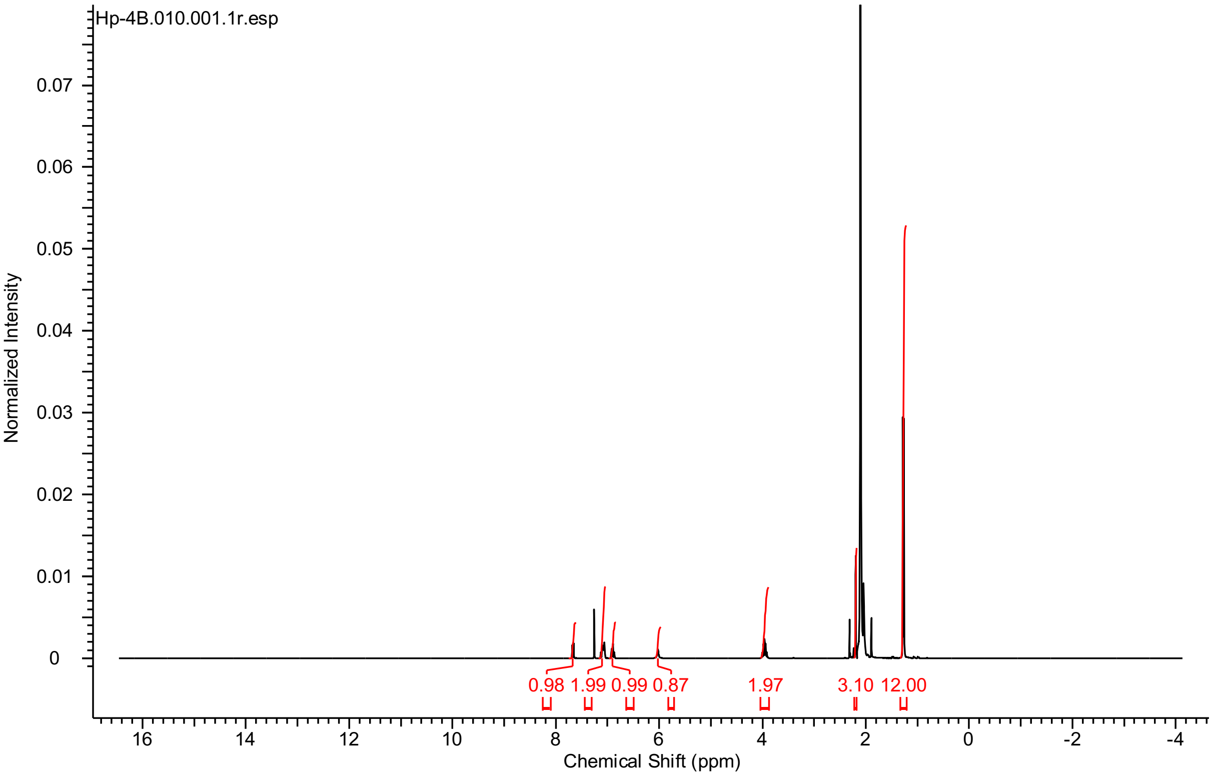

Molecule 5

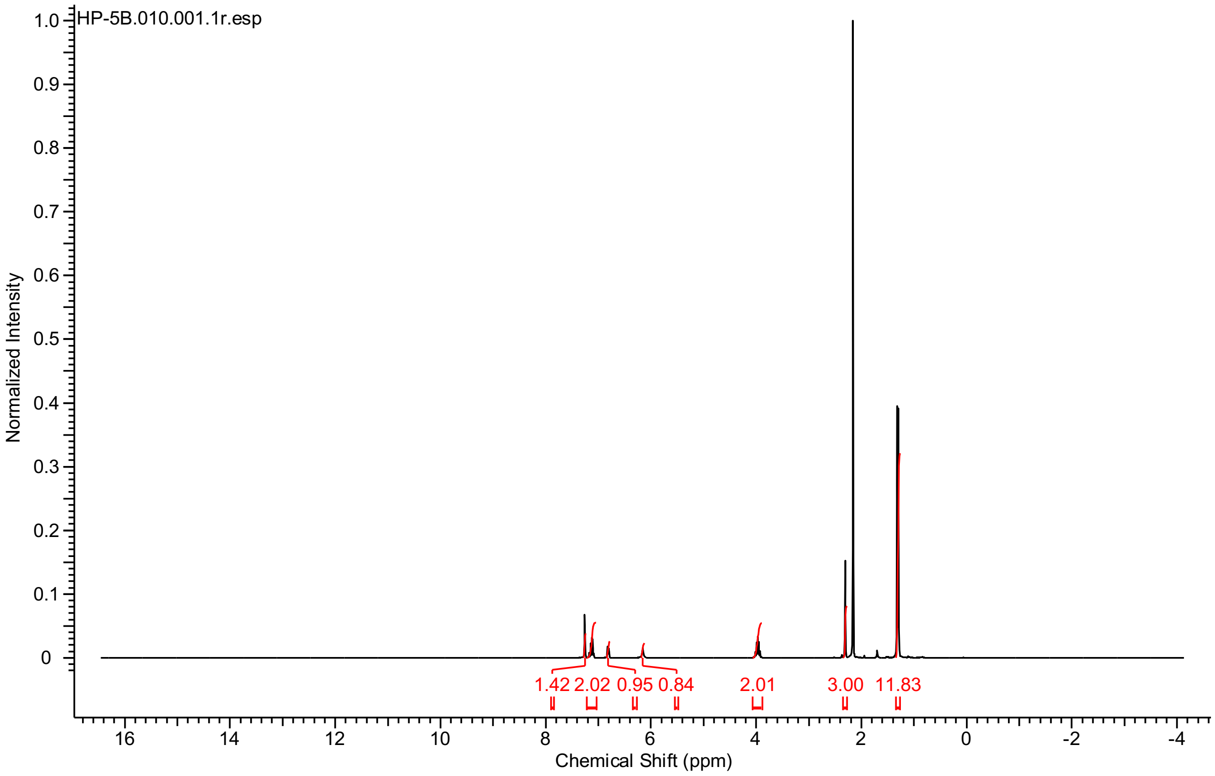

Molecule 6

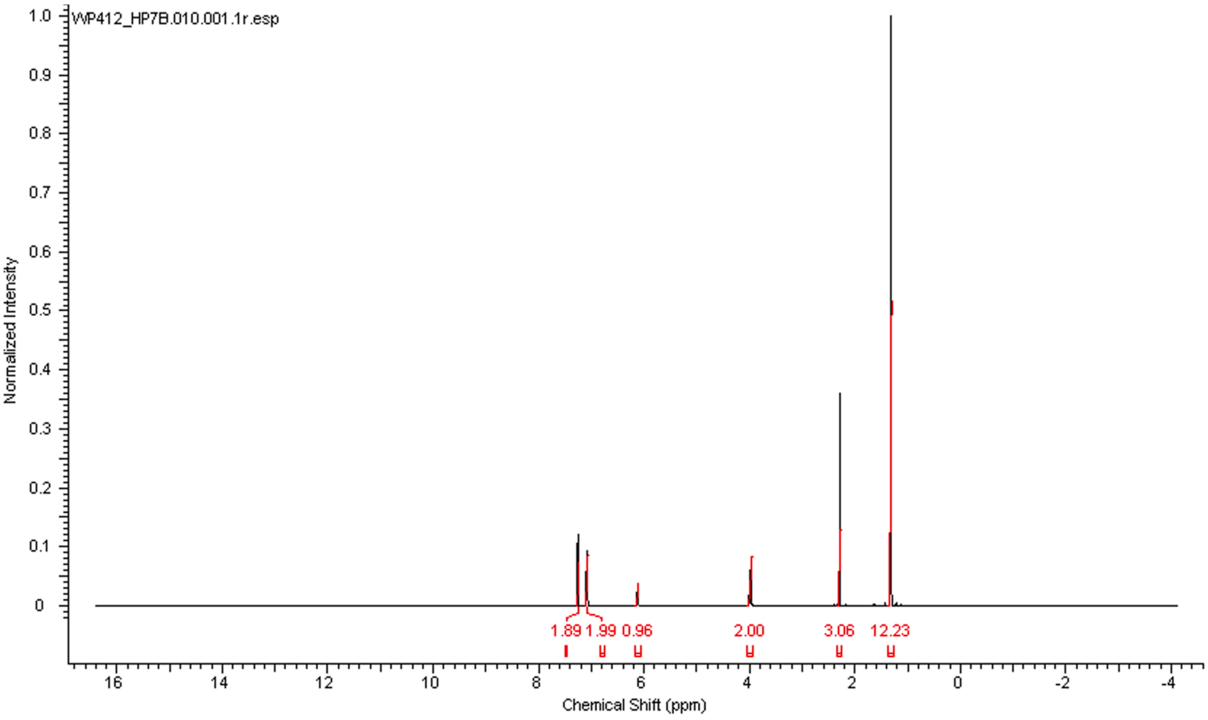

Figure 6 Gels

To form the gels shown in the manuscript **Figure 6** (a), (b) and (c) – 5 wt% of the gelator was added to 1-2 mL of pump oil on the surface of 5 mL of either de-ionised or sea water. The oil layer was heated briefly with a heat gun to cause complete dissolution of the gelator, and then allowed to cool to form a solid gel, before inversion. For (d) 120 mL of water was coloured (for contrast) with a few mg of $CuSO_4$ in a 100 mL, B24 neck, round-bottom flask. Next 3 mL of pump oil was added to the surface, followed by 150 mg of gelator **6**, and the mixture heated briefly with a heat gun at the next of the flask to cause dissolution. The same procedure and result were possible with 150 mg of gelator **5**. After a few minutes of cooling, the gel formed, and the flask was inverted. For (e)/(f) 2 mL of engine oil, recovered used from a 2-stroke engine and filtered, was placed in a vial with 100 mg of gelator **5** and heated briefly with a heat gun. The solution and set gel were photographed.

Crystallography Data for 4, 5 and 6

List of Tables

SXD data on *o*-$CH_3C_6H_4NHCON(^iPr)_2$ (molecule 4) at 22 °C.

Table S1a. Crystal data and structure refinement.
Table S1b. Fractional atomic coordinates and *U*(eq) for all atoms.
Table S1c. Anisotropic displacement parameters.
Table S1d. Selected bond lengths.
Table S1e. Selected bond angles.
Table S1f. Selected torsion angles.

SXD data on *m*-$CH_3C_6H_4NHCON(^iPr)_2$ (molecule 5) at –123 °C.

Table S2a. Crystal data and structure refinement.
Table S2b. Fractional atomic coordinates and *U*(eq) for all atoms.
Table S2c. Anisotropic displacement parameters.
Table S2d. Selected bond lengths.
Table S2e. Selected bond angles.
Table S2f. Selected torsion angles.

SXD data on *p*-$CH_3C_6H_4NHCON(^iPr)_2$ (molecule 6) at –123 °C.

Table S3a. Crystal data and structure refinement.
Table S3b. Fractional atomic coordinates and *U*(eq) for all atoms.
Table S3c. Anisotropic displacement parameters.
Table S3d. Selected bond lengths.
Table S3e. Selected bond angles.
Table S3f. Selected torsion angles.

List of Figures

Figure S1. Evidence for incommensurate behaviour in *o*-$CH_3C_6H_4NHCON(^iPr)_2$.
Figure S2. Labelling of the atoms for *o*-, *m*-, and *p*- $CH_3C_6H_4NHCON(^iPr)_2$.

SXD Measurements and Analysis

Single crystals of *o*-, *m*-, and *p*-$C_{17}H_{28}N_2O$ were selected and measured with Cu Kα radiation on a SuperNova, dual source diffractometer equipped with an Atlas CCD detector. The crystals were kept at 150 K during data collection. For *o*-$C_{17}H_{28}N_2O$, further measurements were made at both 200 K and 295 K. Using Olex2[3], the structures was solved with the ShelXT structure solution program using intrinsic phasing and refined with the ShelXL[4] refinement package using least-squares minimization. Hydrogen atoms were constrained with the AFIX command with the exception of H(15) for *m*- and *p*-$C_{17}H_{28}N_2O$. Further details on all three structures are provided in the summary tables below (Tables S1a-f to S3a-f). For *o*-$C_{17}H_{28}N_2O$, only an approximate structure is presented as the structure is incommensurate at all of the temperatures measured (Figure S1). The labelling of the atoms is similar for all three molecules as shown in Figure S2.

[3] O. V. Dolomanov, L. J. Bourhis, R. J. Gildea, J. A. K. Howard, H. Puschmann, *J. Appl. Crystallogr.* **2009**, *42*, 339-341.
[4] G. M. Sheldrick, *Acta Crystallogr.* **2015**, *A71*, 3-8.

Table S1a. Crystal data and structure refinement for *o*-$CH_3C_6H_4NHCON(^iPr)_2$.

Identification code	exp_1087
Empirical formula	$C_{14}H_{22}N_2O$
Formula weight	234.33
Temperature / K	295
Crystal system	orthorhombic
Space group	$Pna2_1$
a / Å	23.0581(13)
b / Å	16.6608(12)
c / Å	15.1185(10)
α / °	90
β / °	90
γ / °	90
Volume / Å^3	5808.0(7)
Z	16
ρ_{calc} / g cm^{-3}	1.072
μ /mm^{-1}	0.53
F(000)	2048
Crystal size / mm^3	0.347 × 0.091 × 0.069
Radiation	CuKα (λ = 1.54184)
2θ range for data collection / °	7.668 to 147.332
Index ranges	$-28 \le h \le 27$, $-20 \le k \le 18$, $-14 \le l \le 18$
Reflections collected	15196
Independent reflections	8659 [R_{int} = 0.0564, R_{sigma} = 0.0527]
Data/restraints/parameters	8659/1/633
Goodness-of-fit on F^2	1.632
Final R indexes [$I \ge 2\sigma(I)$]	R_1 = 0.1681, wR_2 = 0.4151
Final R indexes [all data]	R_1 = 0.2147, wR_2 = 0.4678
Largest diff. peak/hole / e Å^{-3}	1.40/−0.44

Table S1b. Fractional atomic coordinates and equivalent isotropic displacement parameters for *o*-$CH_3C_6H_4NHCON(^iPr)_2$. U_{eq} is defined as ⅓ of the trace of the orthogonalised U_{ij} tensor.

Atom	*x*	*y*	*z*	*U*(eq) / Å^2
O(1A)	0.7207(3)	0.1950(4)	0.0599(5)	0.0670(17)
N(1A)	0.6730(3)	0.2626(4)	0.1675(5)	0.0521(16)
N(2A)	0.6859(4)	0.1238(5)	0.1752(6)	0.071(2)
C(1A)	0.7584(7)	0.4388(9)	0.0951(12)	0.105(5)
C(2A)	0.7458(4)	0.3652(7)	0.1321(8)	0.073(3)
C(3A)	0.6879(3)	0.3396(5)	0.1279(6)	0.0536(19)
C(4A)	0.6450(5)	0.3862(5)	0.0978(8)	0.069(3)
C(5A)	0.6589(8)	0.4602(7)	0.0590(13)	0.113(6)
C(6A)	0.7171(10)	0.4845(8)	0.0587(15)	0.135(8)
C(7A)	0.7926(5)	0.3106(12)	0.1754(11)	0.121(6)
C(8A)	0.6940(3)	0.1927(5)	0.1301(7)	0.055(2)
C(9A)	0.7005(6)	0.0463(7)	0.1301(11)	0.097(4)
C(10A)	0.6678(7)	0.0341(8)	0.0496(12)	0.106(5)
C(11A)	0.7637(7)	0.0372(9)	0.1173(12)	0.117(6)
C(12A)	0.6662(6)	0.1215(9)	0.2678(9)	0.095(4)
C(13A)	0.6073(7)	0.0801(11)	0.2744(12)	0.115(5)
C(14A)	0.7121(7)	0.0836(12)	0.3267(11)	0.124(6)
O(1B)	0.5602(2)	0.2796(5)	0.2481(4)	0.0662(18)
N(1B)	0.5631(3)	0.2893(6)	0.3975(6)	0.073(2)
N(2B)	0.4764(3)	0.2649(4)	0.3228(5)	0.0533(16)
C(1B)	0.6987(6)	0.4041(13)	0.3675(13)	0.129(7)
C(2B)	0.6391(5)	0.3868(10)	0.3615(10)	0.101(5)
C(3B)	0.6219(4)	0.3136(9)	0.3994(7)	0.084(4)
C(4B)	0.6607(5)	0.2617(11)	0.4415(9)	0.094(4)
C(5B)	0.7187(7)	0.2787(16)	0.4476(13)	0.128(7)
C(6B)	0.7362(6)	0.3550(16)	0.4143(14)	0.142(9)
C(7B)	0.5987(7)	0.4434(9)	0.3217(12)	0.108(4)
C(8B)	0.5348(3)	0.2783(5)	0.3192(7)	0.0536(18)
C(9B)	0.4435(4)	0.2530(5)	0.2390(7)	0.059(2)
C(10B)	0.4445(5)	0.3271(8)	0.1810(8)	0.083(3)
C(11B)	0.4622(5)	0.1781(7)	0.1904(10)	0.080(3)
C(12B)	0.4423(4)	0.2659(8)	0.4041(8)	0.077(3)
C(13B)	0.3987(7)	0.3312(13)	0.4102(12)	0.127(6)
C(14B)	0.4124(8)	0.1832(11)	0.4197(12)	0.119(6)
O(1C)	0.5316(4)	0.1930(5)	0.5577(6)	0.090(2)
N(1C)	0.5765(3)	0.2512(5)	0.6696(6)	0.067(2)
N(2C)	0.5673(5)	0.1192(6)	0.6677(8)	0.091(3)
C(1C)	0.4850(8)	0.4334(11)	0.6153(15)	0.130(6)
C(2C)	0.5025(5)	0.3565(9)	0.6388(10)	0.095(4)
C(3C)	0.5582(4)	0.3302(8)	0.6395(8)	0.082(4)
C(4C)	0.6012(6)	0.3849(7)	0.6025(8)	0.083(3)

C(5C)	0.5844(6)	0.4617(7)	0.5695(9)	0.082(3)
C(6C)	0.5284(12)	0.4817(10)	0.5718(16)	0.154(10)
C(7C)	0.4562(6)	0.3041(14)	0.6783(14)	0.146(8)
C(8C)	0.5590(4)	0.1850(7)	0.6269(8)	0.071(3)
C(9C)	0.5520(8)	0.0432(8)	0.6223(12)	0.109(5)
C(10C)	0.4924(9)	0.0369(10)	0.6032(17)	0.150(9)
C(11C)	0.5912(9)	0.0359(8)	0.5391(9)	0.124(7)
C(12C)	0.5857(6)	0.1133(7)	0.7643(10)	0.093(4)
C(13C)	0.5449(9)	0.0736(11)	0.8297(15)	0.146(8)
C(14C)	0.6461(8)	0.0653(12)	0.7750(11)	0.125(6)
O(1D)	0.6896(3)	0.2732(7)	0.7501(5)	0.095(3)
N(1D)	0.6869(3)	0.2826(6)	0.8991(7)	0.076(2)
N(2D)	0.7739(3)	0.2538(6)	0.8227(6)	0.071(2)
C(1D)	0.5472(6)	0.3948(11)	0.8691(12)	0.112(5)
C(2D)	0.6093(5)	0.3769(9)	0.8657(9)	0.093(4)
C(3D)	0.6275(4)	0.3017(7)	0.9034(10)	0.083(3)
C(4D)	0.5889(6)	0.2447(15)	0.9436(8)	0.125(7)
C(5D)	0.5286(7)	0.2793(17)	0.9500(14)	0.141(9)
C(6D)	0.5128(7)	0.3497(14)	0.9133(16)	0.130(7)
C(7D)	0.6478(6)	0.4277(8)	0.8194(14)	0.108(5)
C(8D)	0.7152(4)	0.2725(8)	0.8209(8)	0.074(3)
C(9D)	0.8060(4)	0.2489(6)	0.7400(7)	0.061(2)
C(10D)	0.7918(6)	0.1732(8)	0.6889(11)	0.103(4)
C(11D)	0.7996(6)	0.3247(8)	0.6849(8)	0.088(3)
C(12D)	0.8085(4)	0.2579(9)	0.9066(7)	0.084(4)
C(13D)	0.8396(7)	0.1808(13)	0.9198(13)	0.135(7)
C(14D)	0.8501(8)	0.3323(13)	0.9060(13)	0.139(7)
H(1A)	0.7973	0.4575	0.0955	0.127
H(4A)	0.6057	0.3694	0.1026	0.083
H(5A)	0.6297	0.4931	0.0336	0.135
H(6A)	0.7274	0.5342	0.0322	0.162
H(7AA)	0.7749	0.2793	0.2233	0.181
H(7AB)	0.8238	0.3440	0.1994	0.181
H(7AC)	0.8085	0.2740	0.1308	0.181
H(9A)	0.6885	0.0026	0.1715	0.117
H(10A)	0.6263	0.0320	0.0635	0.159
H(10B)	0.6797	−0.0165	0.0220	0.159
H(10C)	0.6752	0.0786	0.0087	0.159
H(11A)	0.7785	0.0826	0.0829	0.176
H(11B)	0.7715	−0.0130	0.0856	0.176
H(11C)	0.7830	0.0358	0.1751	0.176
H(12A)	0.6609	0.1782	0.2877	0.114
H(13A)	0.5765	0.1206	0.2744	0.172
H(13B)	0.6055	0.0489	0.3294	0.172

H(13C)	0.6021	0.0440	0.2238	0.172
H(14A)	0.7061	0.0254	0.3292	0.187
H(14B)	0.7092	0.1061	0.3864	0.187
H(14C)	0.7507	0.0949	0.3024	0.187
H(15A)	0.6507	0.2606	0.2147	0.063
H(1B)	0.7135	0.4505	0.3388	0.154
H(4B)	0.6464	0.2133	0.4667	0.113
H(5B)	0.7456	0.2419	0.4725	0.153
H(6B)	0.7748	0.3727	0.4245	0.170
H(7BA)	0.5935	0.4303	0.2590	0.162
H(7BB)	0.6142	0.4980	0.3273	0.162
H(7BC)	0.5613	0.4401	0.3521	0.162
H(9B)	0.4021	0.2445	0.2563	0.071
H(10D)	0.4385	0.3749	0.2176	0.124
H(10E)	0.4135	0.3235	0.1368	0.124
H(10F)	0.4821	0.3309	0.1510	0.124
H(11D)	0.4981	0.1889	0.1578	0.120
H(11E)	0.4317	0.1621	0.1487	0.120
H(11F)	0.4688	0.1348	0.2330	0.120
H(12B)	0.4702	0.2741	0.4540	0.092
H(13D)	0.4166	0.3823	0.3930	0.191
H(13E)	0.3844	0.3351	0.4711	0.191
H(13F)	0.3662	0.3195	0.3704	0.191
H(14D)	0.3852	0.1723	0.3714	0.178
H(14E)	0.3913	0.1844	0.4760	0.178
H(14F)	0.4420	0.1409	0.4216	0.178
H(15B)	0.5445	0.2811	0.4475	0.087
H(1C)	0.4470	0.4529	0.6271	0.156
H(4C)	0.6408	0.3692	0.6003	0.099
H(5C)	0.6124	0.4978	0.5464	0.098
H(6C)	0.5165	0.5299	0.5435	0.185
H(7CA)	0.4463	0.2613	0.6365	0.219
H(7CB)	0.4216	0.3365	0.6906	0.219
H(7CC)	0.4705	0.2804	0.7334	0.219
H(9C)	0.5622	−0.0019	0.6629	0.131
H(10G)	0.4739	0.0016	0.6466	0.225
H(10H)	0.4873	0.0146	0.5437	0.225
H(10I)	0.4746	0.0903	0.6059	0.225
H(11G)	0.5670	0.0357	0.4858	0.186
H(11H)	0.6134	−0.0142	0.5421	0.186
H(11I)	0.6180	0.0815	0.5369	0.186
H(12C)	0.5926	0.1693	0.7857	0.112
H(13G)	0.5178	0.1137	0.8530	0.219
H(13H)	0.5674	0.0506	0.8785	0.219

H(13I)	0.5231	0.0310	0.7999	0.219
H(14G)	0.6495	0.0253	0.7277	0.188
H(14H)	0.6469	0.0382	0.8325	0.188
H(14I)	0.6785	0.1032	0.7712	0.188
H(15C)	0.5992	0.2469	0.7161	0.081
H(1D)	0.5323	0.4401	0.8383	0.134
H(4D)	0.5997	0.1926	0.9633	0.150
H(5D)	0.5001	0.2498	0.9817	0.169
H(6D)	0.4738	0.3669	0.9207	0.157
H(7DA)	0.6854	0.4284	0.8494	0.162
H(7DB)	0.6319	0.4823	0.8178	0.162
H(7DC)	0.6527	0.4079	0.7588	0.162
H(9D)	0.8480	0.2452	0.7564	0.074
H(10J)	0.7827	0.1298	0.7305	0.154
H(10K)	0.8253	0.1577	0.6527	0.154
H(10L)	0.7583	0.1830	0.6505	0.154
H(11J)	0.8020	0.3110	0.6219	0.132
H(11K)	0.8307	0.3624	0.6999	0.132
H(11L)	0.7619	0.3496	0.6972	0.132
H(12D)	0.7807	0.2647	0.9568	0.101
H(13J)	0.8195	0.1380	0.8877	0.203
H(13K)	0.8405	0.1678	0.9830	0.203
H(13L)	0.8793	0.1857	0.8976	0.203
H(14J)	0.8751	0.3300	0.8537	0.209
H(14K)	0.8741	0.3317	0.9595	0.209
H(14L)	0.8271	0.3817	0.9045	0.209
H(15D)	0.7064	0.2770	0.9488	0.091

Table S1c. Anisotropic displacement parameters for *o*-$CH_3C_6H_4NHCON(^iPr)_2$. Anisotropic displacement factor exponent has the form: $-2\pi^2[h^2a^{*2}U_{11}+2hka^*b^*U_{12}+\ldots]$.

Atom	U_{11} / Å^2	U_{22} / Å^2	U_{33} / Å^2	U_{23} / Å^2	U_{13} / Å^2	U_{12} / Å^2
O(1A)	0.086(4)	0.057(3)	0.058(4)	0.009(3)	0.021(3)	0.013(3)
N(1A)	0.050(3)	0.055(4)	0.051(4)	0.003(3)	0.013(3)	0.006(3)
N(2A)	0.098(5)	0.066(5)	0.050(5)	0.019(4)	0.024(4)	0.030(4)
C(1A)	0.104(9)	0.086(8)	0.126(13)	−0.044(9)	0.041(9)	−0.041(8)
C(2A)	0.067(5)	0.077(6)	0.076(7)	−0.018(6)	0.013(5)	−0.012(4)
C(3A)	0.046(4)	0.054(4)	0.061(5)	−0.006(4)	0.012(4)	−0.004(3)
C(4A)	0.081(6)	0.051(5)	0.075(7)	0.000(5)	0.009(5)	0.017(4)
C(5A)	0.149(12)	0.050(5)	0.140(14)	0.013(7)	0.062(11)	0.018(7)
C(6A)	0.188(17)	0.052(6)	0.163(18)	−0.023(9)	0.093(15)	−0.026(9)
C(7A)	0.054(5)	0.219(19)	0.090(10)	−0.039(12)	−0.011(6)	0.000(8)
C(8A)	0.056(4)	0.054(4)	0.054(5)	0.004(4)	0.012(4)	0.021(3)
C(9A)	0.122(9)	0.064(6)	0.107(11)	0.021(7)	0.043(9)	0.026(6)
C(10A)	0.122(11)	0.075(7)	0.120(13)	−0.022(8)	0.018(10)	0.010(7)
C(11A)	0.138(11)	0.107(9)	0.107(12)	0.027(9)	0.038(10)	0.071(9)
C(12A)	0.102(8)	0.111(9)	0.072(8)	0.026(7)	0.017(7)	0.037(7)
C(13A)	0.110(10)	0.128(12)	0.105(12)	0.037(10)	0.023(9)	0.010(9)
C(14A)	0.136(11)	0.160(15)	0.077(9)	0.051(11)	−0.028(9)	0.009(10)
O(1B)	0.049(3)	0.101(5)	0.048(4)	−0.012(4)	0.004(3)	−0.006(3)
N(1B)	0.044(3)	0.127(7)	0.047(4)	−0.007(5)	−0.004(3)	−0.009(4)
N(2B)	0.037(3)	0.069(4)	0.054(4)	0.001(4)	−0.006(3)	−0.006(2)
C(1B)	0.083(8)	0.189(17)	0.114(13)	−0.065(13)	0.017(8)	−0.058(10)
C(2B)	0.060(5)	0.144(12)	0.100(10)	−0.072(9)	0.019(6)	−0.038(7)
C(3B)	0.050(4)	0.153(11)	0.047(6)	−0.046(7)	−0.012(4)	−0.012(6)
C(4B)	0.060(6)	0.156(13)	0.067(7)	−0.025(8)	−0.012(5)	0.000(6)
C(5B)	0.072(8)	0.210(20)	0.102(12)	−0.032(14)	−0.024(8)	0.014(11)
C(6B)	0.069(7)	0.230(20)	0.125(15)	−0.090(17)	−0.011(9)	−0.022(11)
C(7B)	0.121(10)	0.101(9)	0.102(11)	−0.024(9)	0.013(10)	−0.022(8)
C(8B)	0.044(3)	0.065(5)	0.052(5)	−0.007(4)	−0.001(4)	0.001(3)
C(9B)	0.050(4)	0.063(5)	0.064(6)	0.002(4)	−0.019(4)	−0.005(3)
C(10B)	0.089(7)	0.096(8)	0.064(7)	0.015(6)	−0.015(6)	0.006(6)
C(11B)	0.072(5)	0.075(6)	0.092(8)	−0.022(6)	−0.001(6)	−0.013(5)
C(12B)	0.041(4)	0.128(9)	0.060(6)	−0.008(7)	0.004(4)	−0.002(4)
C(13B)	0.122(10)	0.167(15)	0.093(11)	−0.013(11)	0.036(10)	0.059(10)
C(14B)	0.123(11)	0.132(12)	0.101(12)	0.044(10)	0.031(9)	−0.022(9)
O(1C)	0.111(5)	0.077(4)	0.082(6)	0.001(4)	−0.034(5)	−0.009(4)
N(1C)	0.050(4)	0.094(6)	0.057(5)	0.010(4)	−0.014(4)	−0.004(3)
N(2C)	0.105(7)	0.069(6)	0.099(9)	−0.022(6)	−0.017(6)	−0.002(5)
C(1C)	0.119(11)	0.110(12)	0.160(19)	0.040(12)	−0.020(12)	0.026(9)
C(2C)	0.078(7)	0.113(10)	0.094(10)	0.007(8)	−0.017(7)	0.008(6)
C(3C)	0.045(4)	0.133(9)	0.070(7)	0.038(7)	−0.007(4)	0.010(5)
C(4C)	0.108(8)	0.082(7)	0.057(6)	−0.011(6)	−0.004(6)	0.009(6)
C(5C)	0.115(8)	0.056(5)	0.075(8)	0.008(5)	−0.013(7)	−0.012(5)
C(6C)	0.260(30)	0.075(9)	0.128(17)	−0.029(10)	−0.072(19)	0.032(13)
C(7C)	0.072(7)	0.230(20)	0.141(16)	−0.022(16)	−0.002(9)	−0.025(10)
C(8C)	0.048(4)	0.103(8)	0.061(6)	0.026(6)	−0.011(4)	−0.010(4)
C(9C)	0.161(13)	0.064(6)	0.103(12)	−0.020(7)	−0.024(10)	0.000(7)

C(10C)	0.160(15)	0.097(10)	0.190(20)	−0.015(12)	−0.085(15)	−0.037(10)
C(11C)	0.250(20)	0.068(7)	0.058(7)	−0.002(6)	0.009(10)	0.074(10)
C(12C)	0.130(10)	0.069(7)	0.081(9)	0.014(6)	−0.024(8)	−0.011(6)
C(13C)	0.186(17)	0.117(13)	0.134(17)	−0.025(13)	0.071(15)	−0.029(12)
C(14C)	0.166(15)	0.135(13)	0.074(10)	0.000(10)	−0.011(10)	−0.016(12)
O(1D)	0.062(4)	0.164(8)	0.058(5)	0.000(5)	−0.015(4)	0.008(5)
N(1D)	0.072(5)	0.094(6)	0.063(5)	0.014(5)	−0.003(5)	−0.004(4)
N(2D)	0.040(3)	0.124(7)	0.049(4)	0.014(5)	0.003(3)	0.002(3)
C(1D)	0.083(7)	0.133(12)	0.119(13)	−0.029(10)	0.038(8)	0.015(8)
C(2D)	0.095(8)	0.112(10)	0.070(8)	−0.017(7)	−0.021(6)	0.031(7)
C(3D)	0.067(5)	0.096(8)	0.086(8)	−0.016(7)	−0.011(6)	−0.004(5)
C(4D)	0.072(7)	0.260(20)	0.042(6)	0.007(9)	0.014(5)	−0.045(9)
C(5D)	0.082(9)	0.220(20)	0.117(14)	−0.043(16)	0.027(9)	−0.057(13)
C(6D)	0.090(10)	0.159(17)	0.143(18)	−0.024(15)	−0.009(11)	0.039(11)
C(7D)	0.089(7)	0.091(8)	0.143(14)	0.029(10)	0.014(9)	0.010(6)
C(8D)	0.058(5)	0.107(8)	0.056(6)	0.003(6)	0.004(5)	−0.002(5)
C(9D)	0.041(4)	0.088(6)	0.055(6)	−0.006(5)	0.001(4)	0.000(3)
C(10D)	0.117(9)	0.094(8)	0.098(10)	−0.037(8)	−0.011(8)	−0.008(7)
C(11D)	0.117(9)	0.102(8)	0.046(6)	0.006(6)	0.002(6)	0.007(7)
C(12D)	0.047(4)	0.166(12)	0.039(5)	0.000(7)	−0.009(4)	−0.002(5)
C(13D)	0.100(9)	0.196(19)	0.109(13)	0.064(14)	−0.025(9)	0.007(11)
C(14D)	0.140(13)	0.181(18)	0.096(12)	−0.016(13)	−0.032(11)	−0.055(12)

Table S1d. Selected bond lengths for *o*-$CH_3C_6H_4NHCON(^iPr)_2$.

Atom — Atom	Length / Å	Atom — Atom	Length / Å
O(1A) — C(8A)	1.227(12)	O(1C) — C(8C)	1.229(13)
N(1A) — C(3A)	1.457(11)	N(1C) — C(3C)	1.454(15)
N(1A) — C(8A)	1.382(10)	N(1C) — C(8C)	1.340(15)
N(2A) — C(8A)	1.348(12)	N(2C) — C(8C)	1.272(15)
N(2A) — C(9A)	1.499(15)	N(2C) — C(9C)	1.483(16)
N(2A) — C(12A)	1.472(15)	N(2C) — C(12C)	1.523(18)
C(1A) — C(2A)	1.38(2)	C(1C) — C(2C)	1.39(2)
C(1A) — C(6A)	1.34(3)	C(1C) — C(6C)	1.44(3)
C(2A) — C(3A)	1.402(12)	C(2C) — C(3C)	1.358(15)
C(2A) — C(7A)	1.56(2)	C(2C) — C(7C)	1.50(2)
C(3A) — C(4A)	1.337(13)	C(3C) — C(4C)	1.458(18)
C(4A) — C(5A)	1.403(16)	C(4C) — C(5C)	1.428(17)
C(5A) — C(6A)	1.40(3)	C(5C) — C(6C)	1.33(3)
C(9A) — C(10A)	1.45(2)	C(9C) — C(10C)	1.41(2)
C(9A) — C(11A)	1.48(2)	C(9C) — C(11C)	1.55(2)
C(12A) — C(13A)	1.53(2)	C(12C) — C(13C)	1.52(2)
C(12A) — C(14A)	1.522(18)	C(12C) — C(14C)	1.61(2)
O(1B) — C(8B)	1.224(11)	O(1D) — C(8D)	1.222(14)
N(1B) — C(3B)	1.417(11)	N(1D) — C(3D)	1.406(13)
N(1B) — C(8B)	1.364(12)	N(1D) — C(8D)	1.362(15)
N(2B) — C(8B)	1.364(9)	N(2D) — C(8D)	1.389(12)
N(2B) — C(9B)	1.491(11)	N(2D) — C(9D)	1.455(12)
N(2B) — C(12B)	1.460(13)	N(2D) — C(12D)	1.500(12)
C(1B) — C(2B)	1.408(16)	C(1D) — C(2D)	1.464(17)
C(1B) — C(6B)	1.38(3)	C(1D) — C(6D)	1.28(3)
C(2B) — C(3B)	1.40(2)	C(2D) — C(3D)	1.439(19)
C(2B) — C(7B)	1.45(2)	C(2D) — C(7D)	1.41(2)
C(3B) — C(4B)	1.40(2)	C(3D) — C(4D)	1.44(2)
C(4B) — C(5B)	1.37(2)	C(4D) — C(5D)	1.51(3)
C(5B) — C(6B)	1.43(3)	C(5D) — C(6D)	1.35(3)
C(9B) — C(10B)	1.514(15)	C(9D) — C(10D)	1.516(15)
C(9B) — C(11B)	1.510(15)	C(9D) — C(11D)	1.520(16)
C(12B) — C(13B)	1.485(19)	C(12D) — C(13D)	1.48(2)
C(12B) — C(14B)	1.559(19)	C(12D) — C(14D)	1.57(2)

Table S1e. Selected bond angles for *o*-$CH_3C_6H_4NHCON(^iPr)_2$.

Atom —	Atom	— Atom	Angle / °	Atom —	Atom	— Atom	Angle / °
C(6A) —	C(1A)	— C(2A)	121.6(12)	C(2C) —	C(1C)	— C(6C)	115.4(16)
C(1A) —	C(2A)	— C(3A)	116.9(12)	C(1C) —	C(2C)	— C(7C)	115.5(14)
C(1A) —	C(2A)	— C(7A)	123.0(12)	C(3C) —	C(2C)	— C(1C)	125.1(15)
C(3A) —	C(2A)	— C(7A)	120.1(11)	C(3C) —	C(2C)	— C(7C)	118.8(14)
C(2A) —	C(3A)	— N(1A)	118.3(8)	N(1C) —	C(3C)	— C(4C)	119.3(9)
C(4A) —	C(3A)	— N(1A)	118.4(7)	C(2C) —	C(3C)	— N(1C)	124.6(11)
C(4A) —	C(3A)	— C(2A)	123.0(9)	C(2C) —	C(3C)	— C(4C)	116.1(12)
C(3A) —	C(4A)	— C(5A)	118.8(11)	C(5C) —	C(4C)	— C(3C)	120.6(12)
C(6A) —	C(5A)	— C(4A)	118.3(16)	C(6C) —	C(5C)	— C(4C)	118.6(15)
C(1A) —	C(6A)	— C(5A)	121.0(13)	C(5C) —	C(6C)	— C(1C)	123.0(16)
C(8A) —	N(1A)	— C(3A)	119.4(7)	C(8C) —	N(1C)	— C(3C)	120.5(9)
O(1A) —	C(8A)	— N(1A)	120.3(8)	O(1C) —	C(8C)	— N(1C)	118.4(10)
O(1A) —	C(8A)	— N(2A)	122.2(7)	O(1C) —	C(8C)	— N(2C)	125.7(11)
N(2A) —	C(8A)	— N(1A)	117.5(8)	N(2C) —	C(8C)	— N(1C)	115.5(9)
C(8A) —	N(2A)	— C(9A)	118.2(8)	C(8C) —	N(2C)	— C(9C)	118.3(12)
C(8A) —	N(2A)	— C(12A)	123.1(9)	C(8C) —	N(2C)	— C(12C)	124.2(10)
C(12A) —	N(2A)	— C(9A)	118.6(9)	C(9C) —	N(2C)	— C(12C)	117.1(11)
C(10A) —	C(9A)	— N(2A)	112.8(11)	N(2C) —	C(9C)	— C(11C)	107.7(13)
C(10A) —	C(9A)	— C(11A)	113.0(13)	C(10C) —	C(9C)	— N(2C)	113.0(14)
C(11A) —	C(9A)	— N(2A)	111.7(13)	C(10C) —	C(9C)	— C(11C)	113.4(17)
N(2A) —	C(12A)	— C(13A)	110.5(13)	N(2C) —	C(12C)	— C(14C)	111.7(12)
N(2A) —	C(12A)	— C(14A)	110.7(11)	C(13C) —	C(12C)	— N(2C)	118.7(14)
C(14A) —	C(12A)	— C(13A)	113.2(13)	C(13C) —	C(12C)	— C(14C)	104.7(13)
C(6B) —	C(1B)	— C(2B)	121(2)	C(6D) —	C(1D)	— C(2D)	120.2(18)
C(1B) —	C(2B)	— C(7B)	121.2(17)	C(3D) —	C(2D)	— C(1D)	116.7(15)
C(3B) —	C(2B)	— C(1B)	115.2(17)	C(7D) —	C(2D)	— C(1D)	120.6(14)
C(3B) —	C(2B)	— C(7B)	123.5(10)	C(7D) —	C(2D)	— C(3D)	122.4(11)
C(2B) —	C(3B)	— N(1B)	120.6(11)	N(1D) —	C(3D)	— C(2D)	117.5(11)
C(4B) —	C(3B)	— N(1B)	116.4(13)	N(1D) —	C(3D)	— C(4D)	118.3(13)
C(4B) —	C(3B)	— C(2B)	123.0(11)	C(4D) —	C(3D)	— C(2D)	124.2(12)
C(5B) —	C(4B)	— C(3B)	121.7(18)	C(3D) —	C(4D)	— C(5D)	110.2(19)
C(4B) —	C(5B)	— C(6B)	115.9(19)	C(6D) —	C(5D)	— C(4D)	123.8(17)
C(1B) —	C(6B)	— C(5B)	122.1(14)	C(1D) —	C(6D)	— C(5D)	124.0(16)
C(8B) —	N(1B)	— C(3B)	121.0(8)	C(8D) —	N(1D)	— C(3D)	122.4(10)
O(1B) —	C(8B)	— N(1B)	122.0(7)	O(1D) —	C(8D)	— N(1D)	121.8(9)
O(1B) —	C(8B)	— N(2B)	120.7(8)	O(1D) —	C(8D)	— N(2D)	119.3(10)
N(1B) —	C(8B)	— N(2B)	117.3(8)	N(1D) —	C(8D)	— N(2D)	118.6(10)
C(8B) —	N(2B)	— C(9B)	119.3(8)	C(8D) —	N(2D)	— C(9D)	119.4(8)
C(8B) —	N(2B)	— C(12B)	124.3(8)	C(8D) —	N(2D)	— C(12D)	121.6(9)
C(12B) —	N(2B)	— C(9B)	116.3(6)	C(9D) —	N(2D)	— C(12D)	117.3(7)
N(2B) —	C(9B)	— C(10B)	112.1(8)	N(2D) —	C(9D)	— C(10D)	111.9(10)
N(2B) —	C(9B)	— C(11B)	112.2(8)	N(2D) —	C(9D)	— C(11D)	112.0(9)
C(11B) —	C(9B)	— C(10B)	112.8(10)	C(10D) —	C(9D)	— C(11D)	113.0(11)
N(2B) —	C(12B)	— C(13B)	115.2(11)	N(2D) —	C(12D)	— C(14D)	110.9(11)
N(2B) —	C(12B)	— C(14B)	110.8(11)	C(13D) —	C(12D)	— N(2D)	109.4(12)
C(13B) —	C(12B)	— C(14B)	109.8(12)	C(13D) —	C(12D)	— C(14D)	112.9(13)

Table S1f. Selected torsion angles for *o*-$CH_3C_6H_4NHCON(^iPr)_2$ showing degree of twist of phenyl ring with respect to the urea group and planarity of the latter.

Atom — Atom — Atom — Atom	Angle / °	Atom — Atom — Atom — Atom	Angle / °
C(8A) — N(1A) — C(3A) — C(4A)	119.4(10)	C(3C) — N(1C) — C(8C) — N(2C)	−166.5(10)
C(8B) — N(1B) — C(3B) — C(4B)	−120.5(12)	C(3D) — N(1D) — C(8D) — N(2D)	180.0(11)
C(8C) — N(1C) — C(3C) — C(4C)	−112.4(12)	C(9A) — N(2A) — C(8A) — N(1A)	171.2(10)
C(8D) — N(1D) — C(3D) — C(4D)	116.4(15)	C(9B) — N(2B) — C(8B) — N(1B)	179.7(8)
C(3A) — N(1A) — C(8A) — N(2A)	171.2(8)	C(9C) — N(2C) — C(8C) — N(1C)	−176.6(12)
C(3B) — N(1B) — C(8B) — N(2B)	−171.8(10)	C(9D) — N(2D) — C(8D) — N(1D)	−175.3(10)

Table S2a. Crystal data and structure refinement for *m*-$CH_3C_6H_4NHCON(^iPr)_2$.

Identification code	xstr0751
Empirical formula	$C_{17}H_{28}N_2O$
Formula weight	276.41
Temperature / K	150
Crystal system	orthorhombic
Space group	$P2_12_12_1$
a / Å	8.82090(10)
b / Å	19.6496(2)
c / Å	20.23180(10)
α / °	90
β / °	90
γ / °	90
Volume / $Å^3$	3506.72(6)
Z	8
ρ_{calc} / g cm^{-3}	1.047
μ /mm^{-1}	0.501
F(000)	1216.0
Crystal size / mm^3	0.203 × 0.068 × 0.058
Radiation	CuKα (λ = 1.54184)
2θ range for data collection / °	9 to 148.424
Index ranges	$-9 \le h \le 10$, $-24 \le k \le 24$, $-25 \le l \le 24$
Reflections collected	48013
Independent reflections	7051 [R_{int} = 0.0215, R_{sigma} = 0.0119]
Data/restraints/parameters	7051/0/379
Goodness-of-fit on F^2	1.033
Final R indexes [$I \ge 2\sigma(I)$]	R_1 = 0.0543, wR_2 = 0.1549
Final R indexes [all data]	R_1 = 0.0576, wR_2 = 0.1601
Largest diff. peak/hole / e $Å^{-3}$	0.66/−0.26

Table S2b. Fractional atomic coordinates and equivalent isotropic displacement parameters for *m*-$CH_3C_6H_4NHCON(^iPr)_2$. U_{eq} is defined as ⅓ of the trace of the orthogonalised U_{ij} tensor.

Atom	*x*	*y*	*z*	*U*(eq) / Å²
O(1A)	0.12638(19)	0.43388(9)	0.55739(9)	0.0397(4)
N(1A)	0.3417(3)	0.40879(13)	0.61452(11)	0.0451(5)
N(2A)	0.3535(2)	0.45855(12)	0.51047(11)	0.0441(5)
C(1A)	0.1128(4)	0.27714(17)	0.7015(2)	0.0632(9)
C(2A)	0.1689(3)	0.31907(14)	0.65085(15)	0.0478(6)
C(3A)	0.2760(3)	0.36800(15)	0.66440(13)	0.0441(6)
C(4A)	0.3304(4)	0.37529(19)	0.72869(14)	0.0594(8)
C(5A)	0.2740(5)	0.3333(2)	0.77818(17)	0.0754(11)
C(6A)	0.1676(5)	0.2852(2)	0.76458(19)	0.0757(12)
C(7A)	0.0056(5)	0.2229(2)	0.6873(2)	0.0832(12)
C(8A)	0.2665(3)	0.43355(12)	0.56060(12)	0.0365(5)
C(9A)	0.2834(4)	0.50064(14)	0.45799(14)	0.0502(6)
C(10A)	0.1773(4)	0.46185(18)	0.41347(14)	0.0559(7)
C(11A)	0.2119(8)	0.56408(18)	0.4865(2)	0.0947(16)
C(12A)	0.5169(3)	0.4435(3)	0.50381(16)	0.0737(12)
C(13A)	0.5493(5)	0.4039(3)	0.4405(2)	0.1018(19)
C(14A)	0.6125(6)	0.5064(4)	0.5114(3)	0.131(3)
O(1B)	0.6224(2)	0.45447(9)	0.6714(9)	0.0436(4)
N(1B)	0.8412(2)	0.41339(11)	0.62696(11)	0.0385(5)
N(2B)	0.8420(3)	0.50811(12)	0.69608(11)	0.0458(5)
C(1B)	0.6255(3)	0.25110(12)	0.61027(12)	0.0378(5)
C(2B)	0.6746(3)	0.31338(12)	0.63560(11)	0.0364(5)
C(3B)	0.7819(3)	0.35229(12)	0.60168(11)	0.0344(5)
C(4B)	0.8400(3)	0.32823(12)	0.54216(12)	0.0377(5)
C(5B)	0.7921(3)	0.26610(13)	0.51723(12)	0.0410(5)
C(6B)	0.6850(3)	0.22800(12)	0.55052(12)	0.0401(5)
C(7B)	0.5151(3)	0.20880(14)	0.64881(15)	0.0483(6)
C(8B)	0.7609(3)	0.45842(12)	0.66551(11)	0.0358(5)
C(9B)	0.7578(4)	0.56156(16)	0.73294(17)	0.0564(7)
C(10B)	0.6555(6)	0.60405(19)	0.6887(2)	0.0849(13)
C(11B)	0.6766(4)	0.53427(19)	0.79356(18)	0.0674(9)
C(12B)	1.0082(4)	0.5107(2)	0.69904(19)	0.0729(12)
C(13B)	1.0702(7)	0.5716(3)	0.6632(4)	0.140(3)
C(14B)	1.0658(5)	0.5063(3)	0.7680(3)	0.115(2)
C(1C)	0.8727(16)	0.7668(5)	0.4009(5)	0.214(6)
C(2C)	0.8185(12)	0.7426(4)	0.3373(4)	0.145(3)
C(3C)	0.7589(11)	0.6748(4)	0.3382(3)	0.133(3)
C(4C)	0.6594(14)	0.6606(5)	0.3919(5)	0.198(5)
C(5C)	0.7540(20)	0.6757(4)	0.4600(4)	0.219(7)
C(6C)	0.8020(20)	0.7478(4)	0.4596(5)	0.211(6)
H(2A)	0.1334	0.3137	0.6079	0.057

H(4A)	0.4035	0.4079	0.7384	0.071
H(5A)	0.3095	0.3381	0.8212	0.091
H(6A)	0.1315	0.2575	0.7984	0.091
H(7AA)	−0.0557	0.2354	0.6501	0.125
H(7AB)	−0.0582	0.2156	0.7251	0.125
H(7AC)	0.0599	0.1818	0.6774	0.125
H(9A)	0.3669	0.5162	0.4298	0.060
H(10D)	0.2276	0.4220	0.3970	0.084
H(10E)	0.1479	0.4902	0.3770	0.084
H(10F)	0.0889	0.4486	0.4380	0.084
H(11D)	0.1163	0.5527	0.5063	0.142
H(11E)	0.1962	0.5967	0.4519	0.142
H(11F)	0.2778	0.5831	0.5194	0.142
H(12A)	0.5435	0.4134	0.5406	0.088
H(13D)	0.4841	0.3648	0.4384	0.153
H(13E)	0.6532	0.3893	0.4403	0.153
H(13F)	0.5309	0.4325	0.4029	0.153
H(14D)	0.5986	0.5353	0.4736	0.196
H(14E)	0.7173	0.4937	0.5148	0.196
H(14F)	0.5826	0.5304	0.5507	0.196
H(15A)	0.4280(40)	0.4229(17)	0.6229(16)	0.047(9)
H(2B)	0.6356	0.3291	0.6755	0.044
H(4B)	0.9110	0.3539	0.5191	0.045
H(5B)	0.8324	0.2500	0.4778	0.049
H(6B)	0.6525	0.1867	0.5330	0.048
H(7BA)	0.5696	0.1776	0.6765	0.072
H(7BB)	0.4518	0.1838	0.6188	0.072
H(7BC)	0.4533	0.2379	0.6757	0.072
H(9B)	0.8350	0.5930	0.7497	0.068
H(10A)	0.7104	0.6174	0.6498	0.127
H(10B)	0.6232	0.6439	0.7122	0.127
H(10C)	0.5685	0.5777	0.6762	0.127
H(11A)	0.5931	0.5062	0.7800	0.101
H(11B)	0.6395	0.5716	0.8195	0.101
H(11C)	0.7460	0.5077	0.8195	0.101
H(12B)	1.0457	0.4704	0.6757	0.087
H(13A)	1.0237	0.5750	0.6205	0.210
H(13B)	1.1779	0.5669	0.6582	0.210
H(13C)	1.0486	0.6120	0.6882	0.210
H(14A)	1.0283	0.5441	0.7932	0.173
H(14B)	1.1746	0.5072	0.7677	0.173
H(14C)	1.0317	0.4645	0.7878	0.173
H(15B)	0.9270(40)	0.4254(15)	0.6153(15)	0.037(7)
H(1CA)	0.9781	0.7532	0.4044	0.256

H(1CB)	0.8714	0.8161	0.3992	0.256
H(2CA)	0.7401	0.7733	0.3217	0.174
H(2CB)	0.9015	0.7445	0.3059	0.174
H(3CA)	0.7049	0.6669	0.2972	0.159
H(3CB)	0.8430	0.6431	0.3398	0.159
H(4CA)	0.6270	0.6134	0.3906	0.238
H(4CB)	0.5703	0.6894	0.3896	0.238
H(5CA)	0.6906	0.6669	0.4982	0.262
H(5CB)	0.8422	0.6463	0.4625	0.262
H(6CA)	0.7137	0.7764	0.4664	0.253
H(6CB)	0.8711	0.7555	0.4961	0.253

Table S2c. Anisotropic displacement parameters for *m*-$CH_3C_6H_4NHCON(^iPr)_2$. Anisotropic displacement factor exponent has the form: $-2\pi^2[h^2a^{*2}U_{11}+2hka^*b^*U_{12}+\ldots]$.

Atom	U_{11} / Å^2	U_{22} / Å^2	U_{33} / Å^2	U_{23} / Å^2	U_{13} / Å^2	U_{12} / Å^2
O(1A)	0.0279(8)	0.0453(9)	0.0463(9)	−0.0004(7)	−0.0003(7)	0.0006(7)
N(1A)	0.0284(11)	0.0660(14)	0.0413(10)	0.0114(10)	−0.0010(8)	−0.0090(10)
N(2A)	0.0347(11)	0.0552(12)	0.0427(11)	0.0125(9)	−0.0046(9)	−0.0095(9)
C(1A)	0.0466(17)	0.0533(16)	0.0900(2)	0.0256(16)	0.0211(16)	0.0127(13)
C(2A)	0.0339(12)	0.0506(14)	0.0593(15)	0.0148(12)	0.0093(11)	0.0084(11)
C(3A)	0.0287(11)	0.0578(14)	0.0463(13)	0.0132(11)	0.0090(10)	0.0104(11)
C(4A)	0.0533(17)	0.082(2)	0.0431(13)	0.0075(13)	0.0101(13)	0.0116(16)
C(5A)	0.080(2)	0.1010(3)	0.0453(16)	0.0192(17)	0.0200(17)	0.018(2)
C(6A)	0.073(2)	0.091(3)	0.064(2)	0.0371(18)	0.0273(19)	0.020(2)
C(7A)	0.071(3)	0.070(2)	0.109(3)	0.0380(2)	−0.0060(2)	−0.0083(19)
C(8A)	0.0313(11)	0.0369(10)	0.0414(11)	0.0021(9)	−0.0015(9)	−0.0054(9)
C(9A)	0.0562(16)	0.0454(13)	0.0494(13)	0.0137(11)	−0.0096(12)	−0.0067(12)
C(10A)	0.0510(16)	0.0711(18)	0.0457(14)	0.0118(12)	−0.0114(12)	−0.0079(15)
C(11A)	0.1620(5)	0.0444(17)	0.0780(2)	0.0107(16)	−0.005(3)	0.019(2)
C(12A)	0.0314(14)	0.136(3)	0.0540(17)	0.041(2)	−0.0011(12)	−0.0147(18)
C(13A)	0.057(2)	0.186(5)	0.062(2)	0.043(3)	0.0198(18)	0.042(3)
C(14A)	0.078(3)	0.205(7)	0.109(3)	0.074(4)	−0.033(3)	−0.090(4)
O(1B)	0.028(8)	0.0518(10)	0.0513(10)	−0.0139(8)	0.0006(7)	0.0035(7)
N(1B)	0.0269(10)	0.0426(10)	0.0464(11)	−0.0138(8)	0.0051(8)	−0.0046(8)
N(2B)	0.0378(12)	0.0460(11)	0.0540(12)	−0.0216(9)	0.0040(9)	−0.0019(9)
C(1B)	0.0290(11)	0.0410(12)	0.0436(12)	0.0012(9)	−0.0012(10)	0.0005(9)
C(2B)	0.0289(11)	0.0427(11)	0.0378(11)	−0.0036(9)	0.0025(9)	0.0016(9)
C(3B)	0.0249(10)	0.0394(11)	0.0390(11)	−0.0082(9)	−0.0013(9)	−0.0001(8)
C(4B)	0.0290(11)	0.0436(12)	0.0410(11)	−0.0087(9)	0.0052(9)	−0.0053(9)
C(5B)	0.0380(13)	0.0462(12)	0.0392(11)	−0.0130(10)	0.0028(10)	−0.0042(10)
C(6B)	0.0359(12)	0.0394(11)	0.0454(12)	−0.0087(9)	−0.0046(10)	−0.0041(10)
C(7B)	0.0452(15)	0.0444(13)	0.0556(15)	0.0043(11)	0.0078(12)	−0.0019(11)
C(8B)	0.0306(12)	0.0389(11)	0.0382(11)	−0.0088(9)	−0.0015(9)	0.0021(9)
C(9B)	0.0532(16)	0.0470(14)	0.0693(18)	−0.0269(13)	0.0065(14)	0.0030(13)
C(10B)	0.101(3)	0.0510(17)	0.103(3)	−0.0143(18)	−0.002(3)	0.0235(19)
C(11B)	0.064(2)	0.0722(19)	0.0663(19)	−0.0338(16)	0.0144(16)	0.0017(17)
C(12B)	0.0400(16)	0.0910(2)	0.088(2)	−0.061(2)	0.0079(15)	−0.0117(16)
C(13B)	0.114(4)	0.144(5)	0.159(5)	−0.081(4)	0.073(4)	−0.081(4)
C(14B)	0.063(2)	0.156(5)	0.125(4)	−0.090(4)	−0.032(3)	0.0330(3)
C(1C)	0.3080(16)	0.161(8)	0.174(9)	−0.056(7)	0.1150(10)	−0.1130(9)
C(2C)	0.179(8)	0.128(5)	0.127(5)	0.020(4)	0.036(6)	−0.015(6)
C(3C)	0.184(7)	0.125(5)	0.089(3)	0.006(3)	0.003(4)	−0.001(5)
C(4C)	0.2530(12)	0.176(8)	0.165(8)	0.070(7)	−0.035(8)	−0.089(9)
C(5C)	0.4300(20)	0.108(5)	0.111(5)	0.019(4)	0.015(9)	−0.054(8)
C(6C)	0.3800(20)	0.110(5)	0.148(7)	−0.040(5)	−0.0060(10)	−0.018(9)

Table S2d. Selected bond lengths for *m*-$CH_3C_6H_4NHCON(^iPr)_2$.

Atom — Atom	Length / Å	Atom — Atom	Length / Å
O(1A) — C(8A)	1.237(3)	N(2B) — C(8B)	1.359(3)
N(1A) — C(3A)	1.413(3)	N(2B) — C(9B)	1.487(3)
N(1A) — C(8A)	1.367(3)	N(2B) — C(12B)	1.468(4)
N(2A) — C(8A)	1.363(3)	C(1B) — C(2B)	1.396(3)
N(2A) — C(9A)	1.481(3)	C(1B) — C(6B)	1.394(4)
N(2A) — C(12A)	1.478(4)	C(1B) — C(7B)	1.500(4)
C(1A) — C(2A)	1.404(4)	C(2B) — C(3B)	1.397(3)
C(1A) — C(6A)	1.376(6)	C(3B) — C(4B)	1.392(3)
C(1A) — C(7A)	1.454(6)	C(4B) — C(5B)	1.387(3)
C(2A) — C(3A)	1.376(4)	C(5B) — C(6B)	1.381(4)
C(3A) — C(4A)	1.394(4)	C(9B) — C(10B)	1.521(6)
C(4A) — C(5A)	1.391(5)	C(9B) — C(11B)	1.518(5)
C(5A) — C(6A)	1.359(7)	C(12B) — C(13B)	1.503(8)
C(9A) — C(10A)	1.506(4)	C(12B) — C(14B)	1.489(7)
C(9A) — C(11A)	1.512(5)	C(1C) — C(2C)	1.452(13)
C(12A) — C(13A)	1.527(7)	C(1C) — C(6C)	1.396(13)
C(12A) — C(14A)	1.506(7)	C(2C) — C(3C)	1.433(11)
O(1B) — C(8B)	1.230(3)	C(3C) — C(4C)	1.425(12)
N(1B) — C(3B)	1.406(3)	C(4C) — C(5C)	1.637(14)
N(1B) — C(8B)	1.376(3)	C(5C) — C(6C)	1.477(12)

Table S2e. Selected bond angles for *m*-$CH_3C_6H_4NHCON(^iPr)_2$.

Atom — Atom — Atom	Angle / °	Atom — Atom — Atom	Angle / °
C(2A) — C(1A) — C(7A)	121.1(4)	C(2B) — C(1B) — C(7B)	119.8(2)
C(6A) — C(1A) — C(2A)	119.1(4)	C(6B) — C(1B) — C(2B)	119.2(2)
C(6A) — C(1A) — C(7A)	119.7(3)	C(6B) — C(1B) — C(7B)	121.0(2)
C(3A) — C(2A) — C(1A)	120.4(3)	C(1B) — C(2B) — C(3B)	120.6(2)
C(2A) — C(3A) — N(1A)	122.4(3)	C(2B) — C(3B) — N(1B)	122.7(2)
C(2A) — C(3A) — C(4A)	119.7(3)	C(4B) — C(3B) — N(1B)	117.9(2)
C(4A) — C(3A) — N(1A)	117.8(3)	C(4B) — C(3B) — C(2B)	119.3(2)
C(5A) — C(4A) — C(3A)	119.2(4)	C(5B) — C(4B) — C(3B)	120.1(2)
C(6A) — C(5A) — C(4A)	120.9(4)	C(6B) — C(5B) — C(4B)	120.6(2)
C(5A) — C(6A) — C(1A)	120.7(3)	C(5B) — C(6B) — C(1B)	120.2(2)
C(8A) — N(1A) — C(3A)	124.9(2)	C(8B) — N(1B) — C(3B)	124.3(2)
O(1A) — C(8A) — N(1A)	121.9(2)	O(1B) — C(8B) — N(1B)	121.7(2)
O(1A) — C(8A) — N(2A)	121.4(2)	O(1B) — C(8B) — N(2B)	121.6(2)
N(2A) — C(8A) — N(1A)	116.7(2)	N(2B) — C(8B) — N(1B)	116.7(2)
C(8A) — N(2A) — C(9A)	120.0(2)	C(8B) — N(2B) — C(9B)	118.2(2)
C(8A) — N(2A) — C(12A)	123.0(2)	C(8B) — N(2B) — C(12B)	124.7(2)
C(12A) — N(2A) — C(9A)	117.0(2)	C(12B) — N(2B) — C(9B)	117.0(2)
N(2A) — C(9A) — C(10A)	113.9(2)	N(2B) — C(9B) — C(10B)	112.9(3)
N(2A) — C(9A) — C(11A)	111.1(3)	N(2B) — C(9B) — C(11B)	113.0(3)
C(10A) — C(9A) — C(11A)	112.7(3)	C(11B) — C(9B) — C(10B)	112.9(3)
N(2A) — C(12A) — C(13A)	111.1(3)	N(2B) — C(12B) — C(13B)	111.8(4)
N(2A) — C(12A) — C(14A)	111.8(5)	N(2B) — C(12B) — C(14B)	112.1(3)
C(14A) — C(12A) — C(13A)	113.7(4)	C(14B) — C(12B) — C(13B)	112.0(4)

Table S2f. Selected torsion angles for *m*-$CH_3C_6H_4NHCON(^iPr)_2$ showing degree of twist of phenyl ring with respect to the urea group and planarity of the latter.

Atom — Atom — Atom — Atom	Angle / °	Atom — Atom — Atom — Atom	Angle / °
C(8A) — N(1A) — C(3A) — C(4A)	−147.0(3)	C(3B) — N(1B) — C(8B) — N(2B)	167.9(2)
C(8B) — N(1B) — C(3B) — C(4B)	150.4(2)	C(9A) — N(2A) — C(8A) — N(1A)	−165.6(2)
C(3A) — N(1A) — C(8A) — N(2A)	−165.6(3)	C(9B) — N(2B) — C(8B) — N(1B)	174.3(3)

Table S3a. Crystal data and structure refinement for *p*-$CH_3C_6H_4NHCON(^iPr)_2$.

Identification code	xstr0776
Empirical formula	$C_{14}H_{22}N_2O$
Formula weight	234.33
Temperature / K	150
Crystal system	monoclinic
Space group	$P2_1/c$
a / Å	12.9354(2)
b / Å	11.9200(2)
c / Å	27.3126(4)
α / °	90
β / °	93.3110(10)
γ / °	90
Volume / $Å^3$	4204.30(11)
Z	12
ρ_{calc} / g cm^{-3}	1.111
μ /mm^{-1}	0.549
F(000)	1536
Crystal size / mm^3	0.481 × 0.048 × 0.04
Radiation	Cu Kα (λ = 1.54184)
2θ range for data collection / °	6.844 to 147.238
Index ranges	$-16 \le h \le 10$, $-14 \le k \le 13$, $-33 \le l \le 33$
Reflections collected	29459
Independent reflections	8377 [R_{int} = 0.0309, R_{sigma} = 0.0261]
Data/restraints/parameters	8377/0/487
Goodness-of-fit on F^2	1.027
Final R indexes [$I \ge 2\sigma(I)$]	R_1 = 0.0427, wR_2 = 0.1074
Final R indexes [all data]	R_1 = 0.0524, wR_2 = 0.1152
Largest diff. peak/hole / e $Å^{-3}$	0.34/−0.28

Table S3b. Fractional atomic coordinates and equivalent isotropic displacement parameters for *p*-$CH_3C_6H_4NHCON(^iPr)_2$. U_{eq} is defined as ⅓ of the trace of the orthogonalised U_{ij} tensor.

Atom	*x*	*y*	*z*	*U*(eq) / Å^2
O(1A)	0.11431(7)	0.22370(8)	0.29895(3)	0.030(2)
N(1A)	−0.01075(8)	0.14726(9)	0.34413(4)	0.026(2)
N(2A)	−0.05220(9)	0.27134(11)	0.27942(5)	0.036(3)
C(1A)	0.20130(10)	−0.05099(11)	0.38308(5)	0.033(3)
C(2A)	0.14426(10)	0.02567(11)	0.35433(5)	0.031(3)
C(3A)	0.05412(9)	0.07205(10)	0.37145(5)	0.026(2)
C(4A)	0.02327(11)	0.03809(13)	0.41714(5)	0.035(3)
C(5A)	0.08143(12)	−0.03911(13)	0.44495(6)	0.041(3)
C(6A)	0.17214(11)	−0.08443(12)	0.42878(5)	0.035(3)
C(7A)	0.23594(14)	−0.16705(14)	0.45950(7)	0.049(4)
C(8A)	0.02153(9)	0.21528(10)	0.30701(5)	0.026(2)
C(9A)	−0.02082(11)	0.33957(12)	0.23752(5)	0.034(3)
C(10A)	0.04603(14)	0.43985(14)	0.25313(6)	0.049(4)
C(11A)	0.02761(13)	0.27143(15)	0.19779(6)	0.045(4)
C(12A)	−0.16497(12)	0.26204(18)	0.28498(7)	0.057(5)
C(13A)	−0.21230(15)	0.3767(2)	0.29364(7)	0.072(7)
C(14A)	−0.21918(13)	0.20137(18)	0.24121(10)	0.071(7)
O(1B)	0.45267(6)	0.38282(8)	0.37522(3)	0.0283(19)
N(1B)	0.31663(8)	0.33507(10)	0.32206(4)	0.030(2)
N(2B)	0.28917(8)	0.41280(10)	0.39853(4)	0.030(2)
C(1B)	0.51769(10)	0.32455(13)	0.23006(5)	0.033(3)
C(2B)	0.46619(10)	0.35851(12)	0.27073(5)	0.031(3)
C(3B)	0.37481(9)	0.30504(11)	0.28190(5)	0.027(3)
C(4B)	0.33610(10)	0.21957(11)	0.25116(5)	0.030(3)
C(5B)	0.38872(10)	0.18725(12)	0.21078(5)	0.033(3)
C(6B)	0.48081(10)	0.23887(13)	0.19946(5)	0.034(3)
C(7B)	0.53710(13)	0.20299(16)	0.15509(6)	0.048(4)
C(8B)	0.35791(9)	0.37746(10)	0.36606(4)	0.025(2)
C(9B)	0.32580(10)	0.43797(11)	0.44977(5)	0.029(3)
C(10B)	0.37605(12)	0.33641(13)	0.47537(5)	0.037(3)
C(11B)	0.39304(11)	0.54248(12)	0.45439(5)	0.035(3)
C(12B)	0.17682(10)	0.42424(14)	0.38684(5)	0.036(3)
C(13B)	0.11597(12)	0.34055(15)	0.41550(7)	0.048(4)
C(14B)	0.14088(12)	0.54425(15)	0.39447(6)	0.046(4)
O(1C)	0.79550(7)	0.16277(8)	0.40111(4)	0.033(2)
N(1C)	0.66438(8)	0.28967(9)	0.40538(4)	0.029(2)
N(2C)	0.63059(8)	0.09866(9)	0.39265(5)	0.032(2)
C(1C)	0.87976(9)	0.47980(11)	0.44844(5)	0.030(3)
C(2C)	0.82464(10)	0.38137(11)	0.43980(5)	0.029(3)
C(3C)	0.72679(9)	0.38496(10)	0.41558(4)	0.025(2)
C(4C)	0.68622(9)	0.48939(11)	0.40157(5)	0.028(3)

C(5C)	0.74292(10)	0.58666(11)	0.41036(5)	0.030(3)
C(6C)	0.84169(10)	0.58420(11)	0.43380(5)	0.029(3)
C(7C)	0.90447(11)	0.68978(12)	0.44270(6)	0.039(3)
C(8C)	0.70144(9)	0.18204(11)	0.39963(5)	0.027(3)
C(9C)	0.66686(11)	−0.01854(12)	0.38816(7)	0.043(4)
C(10C)	0.72889(15)	−0.03849(17)	0.34377(9)	0.066(6)
C(11C)	0.72108(14)	−0.06098(14)	0.43618(9)	0.061(5)
C(12C)	0.51744(10)	0.11371(12)	0.39338(6)	0.037(3)
C(13C)	0.46300(14)	0.07468(17)	0.34612(8)	0.057(5)
C(14C)	0.47346(14)	0.05806(17)	0.43739(7)	0.054(4)
H(1A)	0.2626	−0.0816	0.3709	0.040
H(2A)	0.1666	0.0465	0.3231	0.037
H(4A)	−0.0382	0.0681	0.4294	0.042
H(5A)	0.0585	−0.0615	0.4759	0.049
H(7AA)	0.2035	−0.2413	0.4571	0.074
H(7AB)	0.2400	−0.1424	0.4938	0.074
H(7AC)	0.3059	−0.1711	0.4476	0.074
H(9A)	−0.0863	0.3715	0.2220	0.040
H(10A)	0.0132	0.4810	0.2791	0.073
H(10B)	0.0534	0.4895	0.2249	0.073
H(10C)	0.1145	0.4137	0.2654	0.073
H(11A)	0.0966	0.2459	0.2097	0.068
H(11B)	0.0336	0.3182	0.1686	0.068
H(11C)	−0.0161	0.2063	0.1895	0.068
H(12A)	−0.1749	0.2155	0.3148	0.068
H(13A)	−0.1725	0.4148	0.3204	0.108
H(13B)	−0.2842	0.3675	0.3025	0.108
H(13C)	−0.2106	0.4217	0.2637	0.108
H(14A)	−0.2148	0.2473	0.2116	0.106
H(14B)	−0.2921	0.1890	0.2476	0.106
H(14C)	−0.1854	0.1290	0.2364	0.106
H(15A)	−0.0714(13)	0.1588(14)	0.3543(6)	0.030(4)
H(1B)	0.5803	0.3612	0.2230	0.040
H(2B)	0.4932	0.4179	0.2908	0.038
H(4B)	0.2731	0.1833	0.2580	0.036
H(5B)	0.3614	0.1286	0.1904	0.039
H(7BA)	0.6004	0.2476	0.1531	0.071
H(7BB)	0.4922	0.2147	0.1254	0.071
H(7BC)	0.5552	0.1233	0.1580	0.071
H(9B)	0.2624	0.4549	0.4677	0.035
H(10D)	0.3305	0.2711	0.4706	0.056
H(10E)	0.3872	0.3520	0.5105	0.056
H(10F)	0.4427	0.3208	0.4614	0.056
H(11D)	0.4605	0.5271	0.4412	0.052

H(11E)	0.4030	0.5636	0.4890	0.052
H(11F)	0.3590	0.6040	0.4359	0.052
H(12B)	0.1638	0.4063	0.3512	0.043
H(13D)	0.1429	0.2649	0.4104	0.073
H(13E)	0.0428	0.3435	0.4041	0.073
H(13F)	0.1229	0.3592	0.4505	0.073
H(14D)	0.1496	0.5634	0.4294	0.069
H(14E)	0.0677	0.5511	0.3835	0.069
H(14F)	0.1822	0.5955	0.3755	0.069
H(15B)	0.2533(14)	0.3090(15)	0.3216(6)	0.035(4)
H(1C)	0.9462	0.4757	0.4650	0.036
H(2C)	0.8535	0.3116	0.4504	0.035
H(4C)	0.6189	0.4938	0.3858	0.033
H(5C)	0.7139	0.6567	0.4002	0.036
H(7CA)	0.8700	0.7381	0.4658	0.059
H(7CB)	0.9104	0.7297	0.4116	0.059
H(7CC)	0.9737	0.6701	0.4565	0.059
H(9C)	0.6029	−0.0651	0.3830	0.051
H(10G)	0.6933	−0.0041	0.3149	0.099
H(10H)	0.7359	−0.1194	0.3384	0.099
H(10I)	0.7978	−0.0050	0.3493	0.099
H(11G)	0.7895	−0.0259	0.4408	0.092
H(11H)	0.7290	−0.1426	0.4346	0.092
H(11I)	0.6793	−0.0415	0.4638	0.092
H(12C)	0.5042	0.1960	0.3962	0.045
H(13G)	0.4942	0.1110	0.3184	0.085
H(13H)	0.3895	0.0946	0.3460	0.085
H(13I)	0.4699	−0.0069	0.3433	0.085
H(14G)	0.4857	−0.0230	0.4360	0.081
H(14H)	0.3988	0.0725	0.4371	0.081
H(14I)	0.5074	0.0886	0.4675	0.081
H(15C)	0.5986(14)	0.3042(15)	0.3957(6)	0.037(4)

Table S3c. Anisotropic displacement parameters for *p*-$CH_3C_6H_4NHCON(^iPr)_2$. Anisotropic displacement factor exponent has the form: $-2\pi^2[h^2a^{*2}U_{11}+2hka^*b^*U_{12}+\ldots]$.

Atom	U_{11} / Å^2	U_{22} / Å^2	U_{33} / Å^2	U_{23} / Å^2	U_{13} / Å^2	U_{12} / Å^2
O(1A)	0.0230(4)	0.0349(5)	0.0324(5)	0.0034(4)	0.0006(3)	−0.0028(3)
N(1A)	0.0201(5)	0.0309(5)	0.0279(5)	0.0017(4)	0.0005(4)	0.0008(4)
N(2A)	0.0257(5)	0.0471(7)	0.0357(6)	0.0141(5)	0.0020(5)	0.0057(5)
C(1A)	0.0277(6)	0.0302(6)	0.0419(7)	−0.0007(5)	0.0029(5)	0.0039(5)
C(2A)	0.0292(6)	0.0323(6)	0.0310(6)	0.0001(5)	0.0033(5)	0.0017(5)
C(3A)	0.0223(5)	0.0259(6)	0.0280(6)	−0.0013(5)	−0.0021(4)	−0.0018(4)
C(4A)	0.0319(7)	0.0407(7)	0.0332(7)	0.0054(6)	0.0066(5)	0.0083(6)
C(5A)	0.0447(8)	0.0442(8)	0.0347(7)	0.0112(6)	0.0077(6)	0.0088(6)
C(6A)	0.0358(7)	0.0291(6)	0.0395(7)	0.0029(5)	−0.0025(6)	0.0044(5)
C(7A)	0.0519(9)	0.0429(9)	0.0517(10)	0.0112(7)	−0.0010(7)	0.0145(7)
C(8A)	0.0258(6)	0.0272(6)	0.0250(6)	−0.0009(5)	−0.0007(5)	−0.0008(5)
C(9A)	0.0343(7)	0.0377(7)	0.0286(6)	0.0073(5)	−0.0011(5)	0.0017(5)
C(10A)	0.0644(10)	0.0371(8)	0.0435(9)	0.0085(7)	−0.0103(8)	−0.0061(7)
C(11A)	0.0472(9)	0.0574(10)	0.0313(7)	−0.0006(7)	0.0022(6)	0.0026(7)
C(12A)	0.0286(7)	0.0853(13)	0.0561(10)	0.0373(10)	0.0055(7)	0.0145(8)
C(13A)	0.0486(10)	0.1250(2)	0.0412(9)	−0.0109(11)	0.0015(8)	0.0383(12)
C(14A)	0.0301(8)	0.0565(11)	0.1240(2)	0.0113(12)	−0.0151(10)	0.0020(7)
O(1B)	0.0201(4)	0.0360(5)	0.0285(4)	−0.0041(4)	−0.0017(3)	0.0023(3)
N(1B)	0.0198(5)	0.0426(6)	0.0277(5)	−0.0100(5)	0.0020(4)	−0.0027(4)
N(2B)	0.0206(5)	0.0428(6)	0.0252(5)	−0.0078(4)	−0.0007(4)	0.0036(4)
C(1B)	0.0245(6)	0.0449(8)	0.0312(7)	0.0012(6)	0.0034(5)	0.0006(5)
C(2B)	0.0269(6)	0.0373(7)	0.0296(6)	−0.0051(5)	0.0013(5)	−0.0014(5)
C(3B)	0.0224(6)	0.0347(6)	0.0248(6)	−0.0030(5)	0.0004(5)	0.0040(5)
C(4B)	0.0251(6)	0.0368(7)	0.0269(6)	−0.0033(5)	−0.0002(5)	−0.0006(5)
C(5B)	0.0334(7)	0.0378(7)	0.0270(6)	−0.0072(5)	−0.0012(5)	0.0020(5)
C(6B)	0.0309(6)	0.0437(8)	0.0275(6)	−0.0027(6)	0.0028(5)	0.0066(6)
C(7B)	0.0428(8)	0.0639(10)	0.0373(8)	−0.0110(7)	0.0119(7)	0.0027(7)
C(8B)	0.0232(6)	0.0281(6)	0.0244(6)	−0.0016(5)	0.0004(4)	0.0018(4)
C(9B)	0.0269(6)	0.0371(7)	0.0229(6)	−0.0053(5)	0.0000(5)	0.0018(5)
C(10B)	0.0425(8)	0.0404(8)	0.0291(7)	0.0001(6)	−0.0002(6)	0.0024(6)
C(11B)	0.0338(7)	0.0375(7)	0.0318(7)	−0.0072(5)	−0.0021(5)	−0.0004(5)
C(12B)	0.0216(6)	0.0555(9)	0.0312(7)	−0.0143(6)	−0.0022(5)	0.0062(6)
C(13B)	0.0294(7)	0.0525(9)	0.0637(11)	−0.0231(8)	0.0078(7)	−0.0079(6)
C(14B)	0.0344(7)	0.0582(10)	0.0449(9)	0.0011(7)	0.0004(6)	0.0162(7)
O(1C)	0.0226(4)	0.0303(5)	0.0467(6)	−0.0032(4)	0.0038(4)	0.0034(3)
N(1C)	0.0204(5)	0.0255(5)	0.0405(6)	0.0007(4)	0.0002(4)	0.0017(4)
N(2C)	0.0242(5)	0.0235(5)	0.0473(7)	−0.0003(5)	0.0011(5)	0.0018(4)
C(1C)	0.0234(6)	0.0348(7)	0.0314(6)	−0.0028(5)	−0.0020(5)	0.0009(5)
C(2C)	0.0270(6)	0.0285(6)	0.0313(6)	0.0029(5)	−0.0017(5)	0.0028(5)
C(3C)	0.0224(5)	0.0264(6)	0.0258(6)	−0.0004(5)	0.0041(4)	0.0005(4)
C(4C)	0.0240(6)	0.0285(6)	0.0304(6)	−0.0021(5)	−0.0009(5)	0.0038(5)
C(5C)	0.0311(6)	0.0251(6)	0.0338(7)	−0.0013(5)	−0.0001(5)	0.0042(5)
C(6C)	0.0270(6)	0.0296(6)	0.0299(6)	−0.0054(5)	0.0036(5)	−0.0005(5)
C(7C)	0.0351(7)	0.0324(7)	0.0497(9)	−0.0071(6)	−0.0001(6)	−0.0029(6)
C(8C)	0.0250(6)	0.0262(6)	0.0297(6)	0.0004(5)	0.0024(5)	0.0022(5)
C(9C)	0.0313(7)	0.0243(6)	0.0728(11)	−0.0074(7)	0.0037(7)	0.0011(5)

C(10C)	0.0481(10)	0.0540(10)	0.0980(16)	−0.0382(11)	0.0220(10)	−0.0082(8)
C(11C)	0.0484(9)	0.0328(8)	0.1005(16)	0.0152(9)	−0.0105(10)	0.0063(7)
C(12C)	0.0248(6)	0.0273(6)	0.0596(9)	0.0003(6)	0.0013(6)	−0.0003(5)
C(13C)	0.0417(9)	0.0629(11)	0.0633(11)	0.0142(9)	−0.0150(8)	−0.0061(8)
C(14C)	0.0421(8)	0.0597(11)	0.0624(11)	−0.0051(9)	0.0150(8)	−0.0065(8)

Table S3d. Selected bond lengths for *p*-$CH_3C_6H_4NHCON(^iPr)_2$.

Atom — Atom	Length / Å	Atom — Atom	Length / Å
O(1A) — C(8A)	1.2368(16)	C(3B) — C(4B)	1.3946(18)
N(1A) — C(3A)	1.4116(16)	C(4B) — C(5B)	1.3838(19)
N(1A) — C(8A)	1.3813(17)	C(5B) — C(6B)	1.391(2)
N(2A) — C(8A)	1.3567(17)	C(6B) — C(7B)	1.511(2)
N(2A) — C(9A)	1.4797(18)	C(9B) — C(10B)	1.5246(19)
N(2A) — C(12A)	1.4795(19)	C(9B) — C(11B)	1.5204(19)
C(1A) — C(2A)	1.3888(19)	C(12B) — C(13B)	1.516(2)
C(1A) — C(6A)	1.383(2)	C(12B) — C(14B)	1.522(2)
C(2A) — C(3A)	1.3952(18)	O(1C) — C(8C)	1.2366(15)
C(3A) — C(4A)	1.3920(19)	N(1C) — C(3C)	1.4119(16)
C(4A) — C(5A)	1.387(2)	N(1C) — C(8C)	1.3817(17)
C(5A) — C(6A)	1.387(2)	N(2C) — C(8C)	1.3580(17)
C(6A) — C(7A)	1.508(2)	N(2C) — C(9C)	1.4810(17)
C(9A) — C(10A)	1.522(2)	N(2C) — C(12C)	1.4758(17)
C(9A) — C(11A)	1.519(2)	C(1C) — C(2C)	1.3860(19)
C(12A) — C(13A)	1.522(3)	C(1C) — C(6C)	1.3887(19)
C(12A) — C(14A)	1.532(3)	C(2C) — C(3C)	1.3947(18)
O(1B) — C(8B)	1.2384(15)	C(3C) — C(4C)	1.3958(18)
N(1B) — C(3B)	1.4118(16)	C(4C) — C(5C)	1.3855(18)
N(1B) — C(8B)	1.3824(16)	C(5C) — C(6C)	1.3959(19)
N(2B) — C(8B)	1.3586(16)	C(6C) — C(7C)	1.5098(19)
N(2B) — C(9B)	1.4821(16)	C(9C) — C(10C)	1.511(3)
N(2B) — C(12B)	1.4761(16)	C(9C) — C(11C)	1.537(3)
C(1B) — C(2B)	1.3881(19)	C(12C) — C(13C)	1.508(2)
C(1B) — C(6B)	1.387(2)	C(12C) — C(14C)	1.512(2)
C(2B) — C(3B)	1.3922(19)		

Table S3e. Selected bond angles for *p*-$CH_3C_6H_4NHCON(^iPr)_2$.

Atom — Atom — Atom	Angle / °	Atom — Atom — Atom	Angle / °
C(6A)— C(1A) — C(2A)	122.41(12)	O(1B) — C(8B) — N(2B)	121.99(11)
C(1A)— C(2A) — C(3A)	119.89(13)	N(2B) — C(8B) — N(1B)	116.51(10)
C(2A)— C(3A) — N(1A)	123.87(12)	C(8B) — N(2B) — C(9B)	119.57(10)
C(4A)— C(3A) — N(1A)	117.79(11)	C(8B) — N(2B) — C(12B)	124.11(11)
C(4A)— C(3A) — C(2A)	118.27(12)	C(12B) — N(2B) — C(9B)	116.28(10)
C(5A)— C(4A) — C(3A)	120.62(13)	N(2B) — C(9B) — C(10B)	112.15(11)
C(6A)— C(5A) — C(4A)	121.75(14)	N(2B) — C(9B) — C(11B)	113.23(11)
C(1A)— C(6A) — C(5A)	117.06(13)	C(11B) — C(9B) — C(10B)	112.70(11)
C(1A)— C(6A) — C(7A)	121.41(13)	N(2B) — C(12B) — C(13B)	111.19(13)
C(5A)— C(6A) — C(7A)	121.53(14)	N(2B) — C(12B) — C(14B)	111.21(12)
C(8A)— N(1A) — C(3A)	124.41(10)	C(13B) — C(12B) — C(14B)	112.08(12)
O(1A)— C(8A) — N(1A)	121.06(11)	C(2C) — C(1C) — C(6C)	122.63(11)
O(1A)— C(8A) — N(2A)	121.28(12)	C(1C) — C(2C) — C(3C)	119.91(12)
N(2A)— C(8A) — N(1A)	117.65(11)	C(2C) — C(3C) — N(1C)	124.18(11)
C(8A)— N(2A) — C(9A)	119.01(11)	C(2C) — C(3C) — C(4C)	118.29(11)
C(8A)— N(2A) — C(12A)	124.87(12)	C(4C) — C(3C) — N(1C)	117.51(11)
C(12A)— N(2A) — C(9A)	115.88(11)	C(5C) — C(4C) — C(3C)	120.80(11)
N(2A)— C(9A) — C(10A)	113.08(12)	C(4C) — C(5C) — C(6C)	121.55(12)
N(2A)— C(9A) — C(11A)	113.66(12)	C(1C) — C(6C) — C(5C)	116.80(12)
C(11A)— C(9A) — C(10A)	111.65(13)	C(1C) — C(6C) — C(7C)	121.48(12)
N(2A)— C(12A)— C(13A)	110.88(17)	C(5C) — C(6C) — C(7C)	121.73(12)
N(2A)— C(12A)— C(14A)	111.41(16)	C(8C) — N(1C) — C(3C)	124.83(10)
C(13A)— C(12A)— C(14A)	112.05(14)	O(1C) — C(8C) — N(1C)	121.06(12)
C(6B)— C(1B) — C(2B)	122.20(13)	O(1C) — C(8C) — N(2C)	121.56(11)
C(1B)— C(2B) — C(3B)	119.64(12)	N(2C) — C(8C) — N(1C)	117.37(11)
C(2B)— C(3B) — N(1B)	123.47(12)	C(8C) — N(2C) — C(9C)	119.18(11)
C(2B)— C(3B) — C(4B)	118.85(12)	C(8C) — N(2C) — C(12C)	124.81(11)
C(4B)— C(3B) — N(1B)	117.65(11)	C(12C) — N(2C) — C(9C)	115.78(11)
C(5B)— C(4B) — C(3B)	120.47(12)	N(2C) — C(9C) — C(10C)	113.60(15)
C(4B)— C(5B) — C(6B)	121.39(13)	N(2C) — C(9C) — C(11C)	111.78(14)
C(1B)— C(6B) — C(5B)	117.44(12)	C(10C) — C(9C) — C(11C)	113.31(16)
C(1B)— C(6B) — C(7B)	121.80(13)	N(2C) — C(12C) — C(13C)	111.43(14)
C(5B)— C(6B) — C(7B)	120.76(13)	N(2C) — C(12C) — C(14C)	112.11(13)
C(8B)— N(1B) — C(3B)	124.87(11)	C(13C) — C(12C) — C(14C)	111.49(14)
O(1B)— C(8B) — N(1B)	121.51(11)		

Table S3f. Selected torsion angles for *p*-$CH_3C_6H_4NHCON(^iPr)_2$ showing degree of twist of phenyl ring with respect to the urea group and planarity of the latter.

Atom — Atom — Atom — Atom	Angle / °	Atom — Atom — Atom — Atom	Angle / °
C(8A) — N(1A) — C(3A) — C(4A)	158.59(13)	C(3C) — N(1C) — C(8C) — N(2C)	176.40(12)
C(8B) — N(1B) — C(3B) — C(4B)	150.48(13)	C(9A) — N(2A) — C(8A) — N(1A)	−176.40(12)
C(8C) — N(1C) — C(3C) — C(4C)	155.13(12)	C(9B) — N(2B) — C(8B) — N(1B)	167.94(12)
C(3A) — N(1A) — C(8A) — N(2A)	170.86(12)	C(9C) — N(2C) — C(8C) — N(1C)	−177.74(13)
C(3B) — N(1B) — C(8B) — N(2B)	173.19(12)		

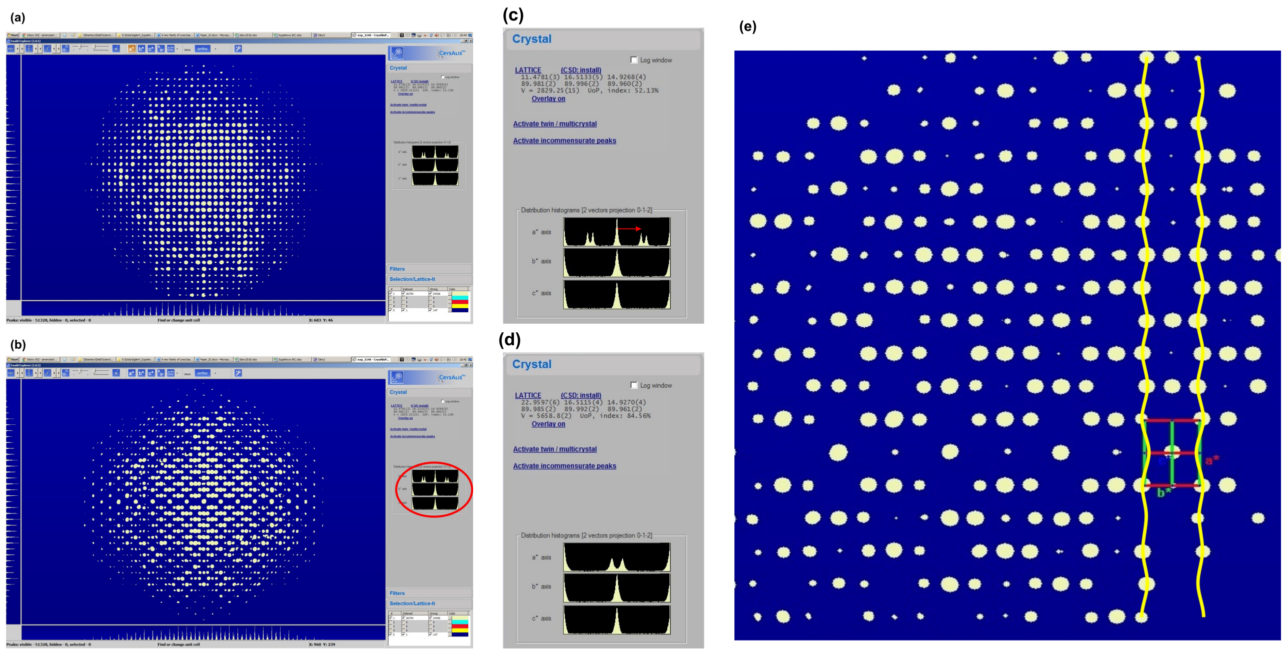

Figure S1. Plots of reciprocal space using the Explorer option within CrysAlisPro showing projection views of a partial data set collected on a crystal of *o*-$CH_3C_6H_4NHCON(^iPr)_2$ at –123 °C. Seen down **a***, the position of the spots looks normal (commensurate) as in view (a) but viewed down **c*** as in view (b), the incommensurate nature of the crystal structure is apparent. This is evident from the distribution histogram in seen in Explorer highlighted by the red ellipse in view (b) where a pair of satellite peaks in the distribution is seen with respect to *a**. An enlarged view of the distribution is shown in (c): the arrow in red highlights the incommensurate vector along *a**. If the lattice parameter *a* is doubled as shown in (d), the position of the satellite peaks will lie close to the lattice points of the reciprocal space cell (e). A wave with amplitude along *a** can be used to describe the off-centre position of the spots. Alternatively, the data can be integrated using a large integration sphere to yield an approximate structure.

Molecule 4

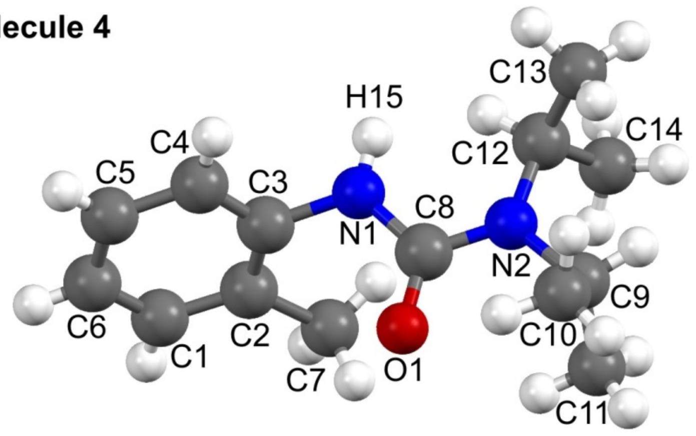

Molecule 5

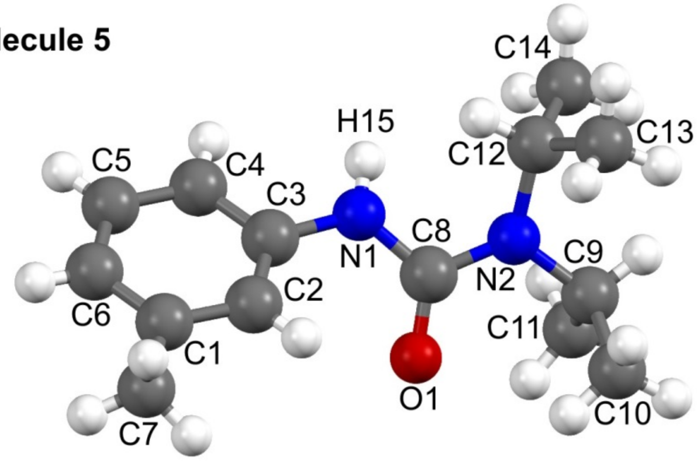

Molecule 6

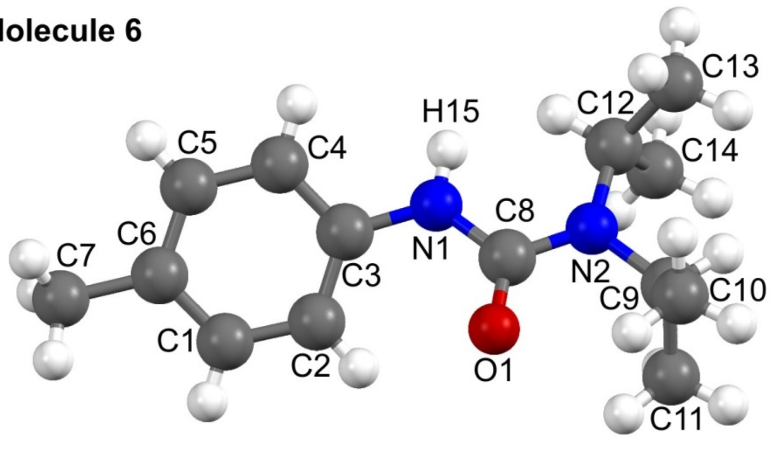

Figure S2. Labelling of the atoms used for the 3 crystal structures. H atom labels are assigned according to the number of the heavy atom to which they are attached with the exception of H15 which is attached to N1.

Rheology of gels of 4, 5 and 6 at 2% w/v in pump oil

An AR-G2 controlled stress rheometer from TA Instruments fitted with a Peltier plate was used. Initially, the sample was melted and approximately 1 mL was placed on the rheometer, which was closed to a gap of 300 µm with a 60 mm flat plate geometry. The sample was then heated to 70 °C and sheared at 1 Pa for 2 minutes in order to break down any SAFIN structures, minimizing the effects of sample history. The sample was then cooled down to 25 °C at a rate of 5 °C min^{-1} followed by a hold at 25 ^{o}C for 10 minutes. During the cooling ramp the plate geometry was oscillated at 2π rad s^{-1}, 0.3 Pa. A time sweep was then conducted for 10 minutes using 0.3 Pa at 2π rad s^{-1}. This time sweep captured the network formation processes and we observed that all samples had stopped evolving before any other measurements were taken.

Each sample was then subjected to a frequency sweep or stress sweep. These experiments measured the shear storage and loss moduli, G′ and G′′ respectively, which describe the solid- and liquid-like nature of the samples. The frequency sweep was used to ensure we were working in a region where the materials showed a strong gel structure (ie. G′>G′′ and G′ was frequency independent). The frequency sweep was performed using a stress of 0.3 Pa (10 Pa for **5**) between 0.1 and 100 Hz. We used oscillatory stress sweep experiments to identify the linear viscoelastic region and then to measure the samples to breakdown, with oscillatory stress amplitudes started from 0.1 Pa and increased up to 1000 Pa, with a frequency of 2π rad s^{-1}.

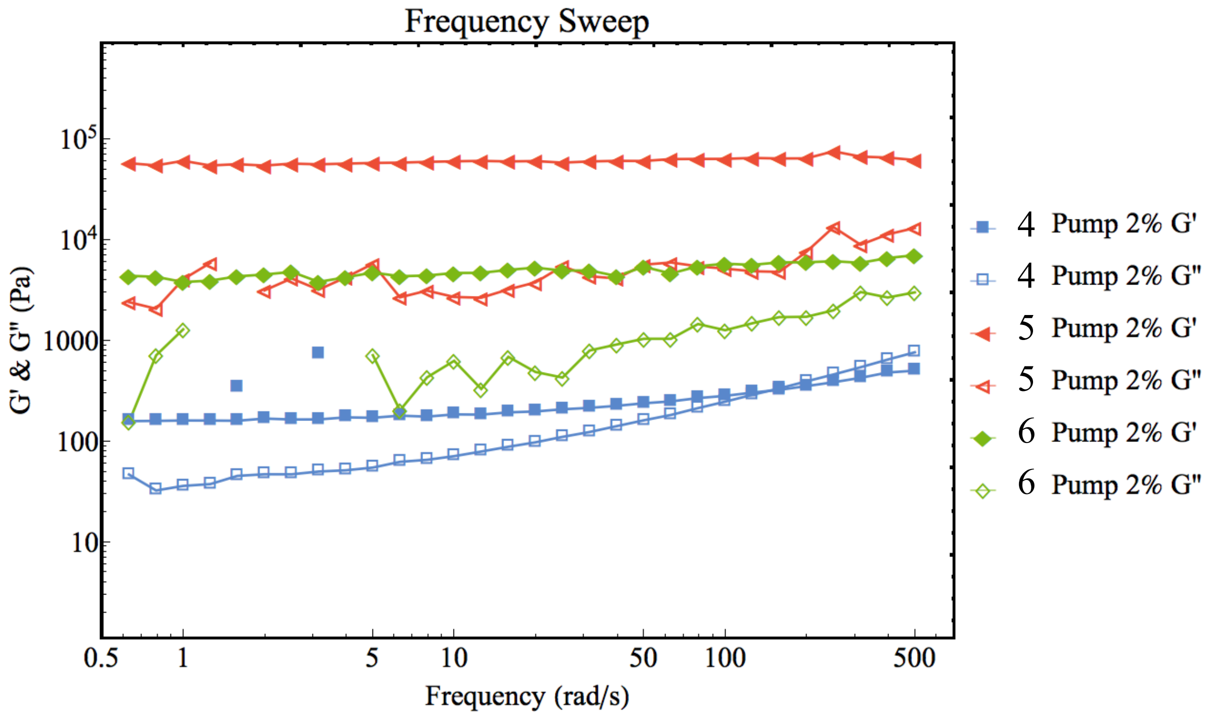

Figure S3. Frequency Sweep (G' and G") on 2% pump-oil gels of **4**, **5** and **6**

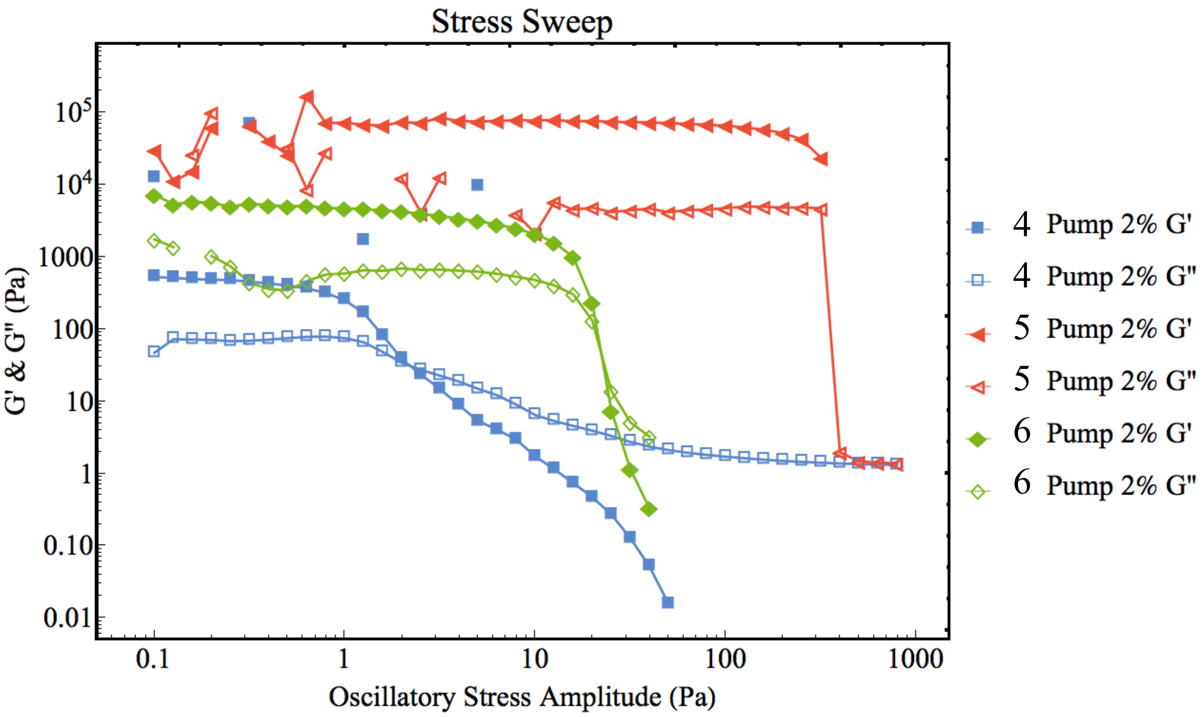

Figure S4. Stress Sweep (G' and G'') on 2% pump-oil gels of **4**, **5** and **6**

DSC of gels of 4, 5 and 6 at 2% w/v in pump oil

We used a Q20 heat flux DSC from TA Instruments which was calibrated with an indium sample at 10 °C min^{-1}. Approximately 25 mg of sample was placed into stainless steel pans, which were subsequently hermitically sealed and placed into the furnace of the DSC. At the beginning of the experiment, the sample was heated at 100 °C for 1 minute in order to destroy any crystal memory. Next, the sample was cooled at 5 °C min^{-1} to 25 °C followed by a heating ramp back at 5 °C min^{-1} to bring the sample to 100 °C.

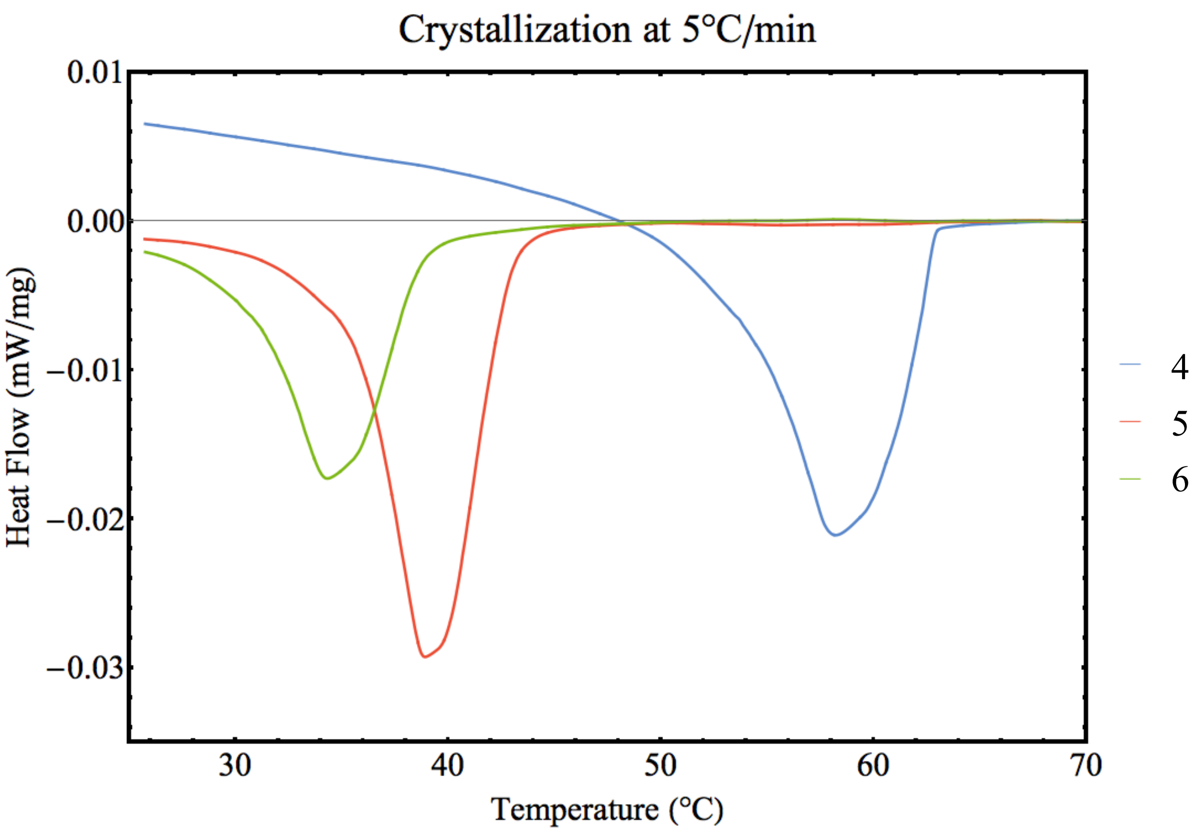

Figure S5. DSC cooling at 5 ^{o}C min^{-1} on 2% pump-oil gels of **4**, **5** and **6**

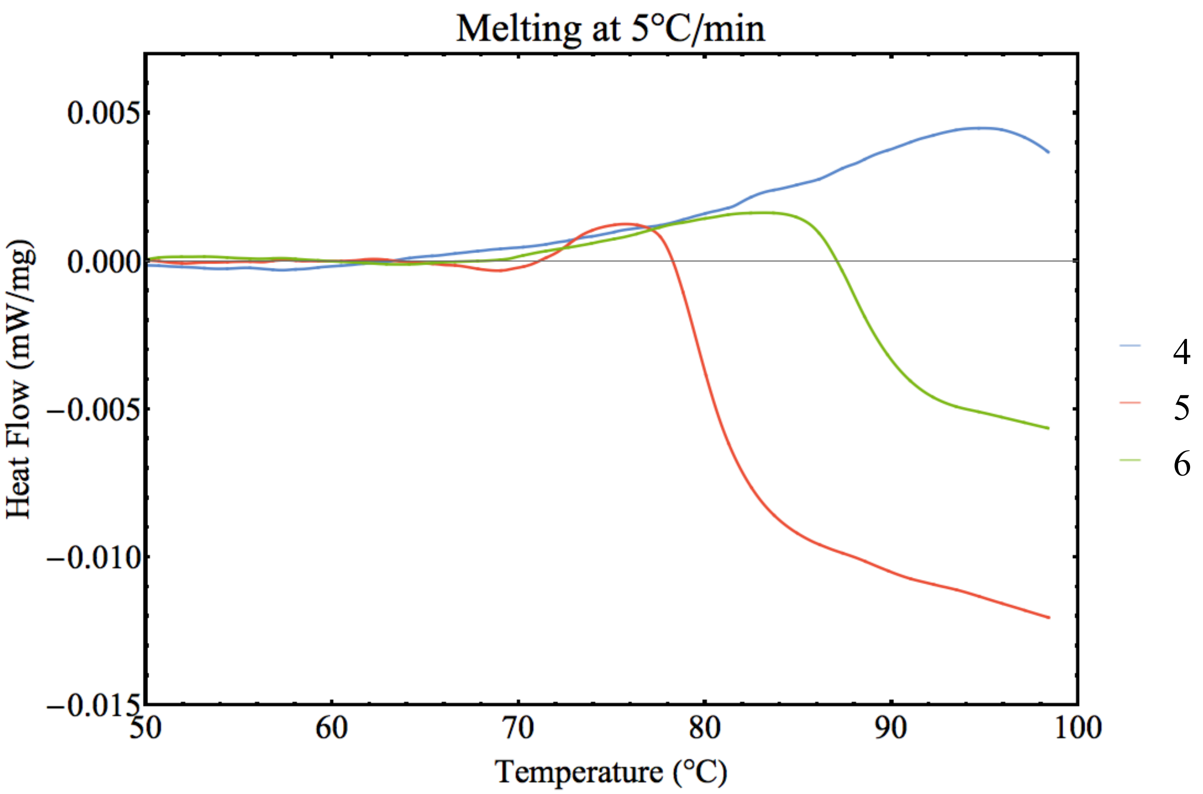

Figure S6. DSC heating at 5 ^{o}C min^{-1} on 2% pump-oil gels of **4**, **5** and **6**

Additional SEM imagery

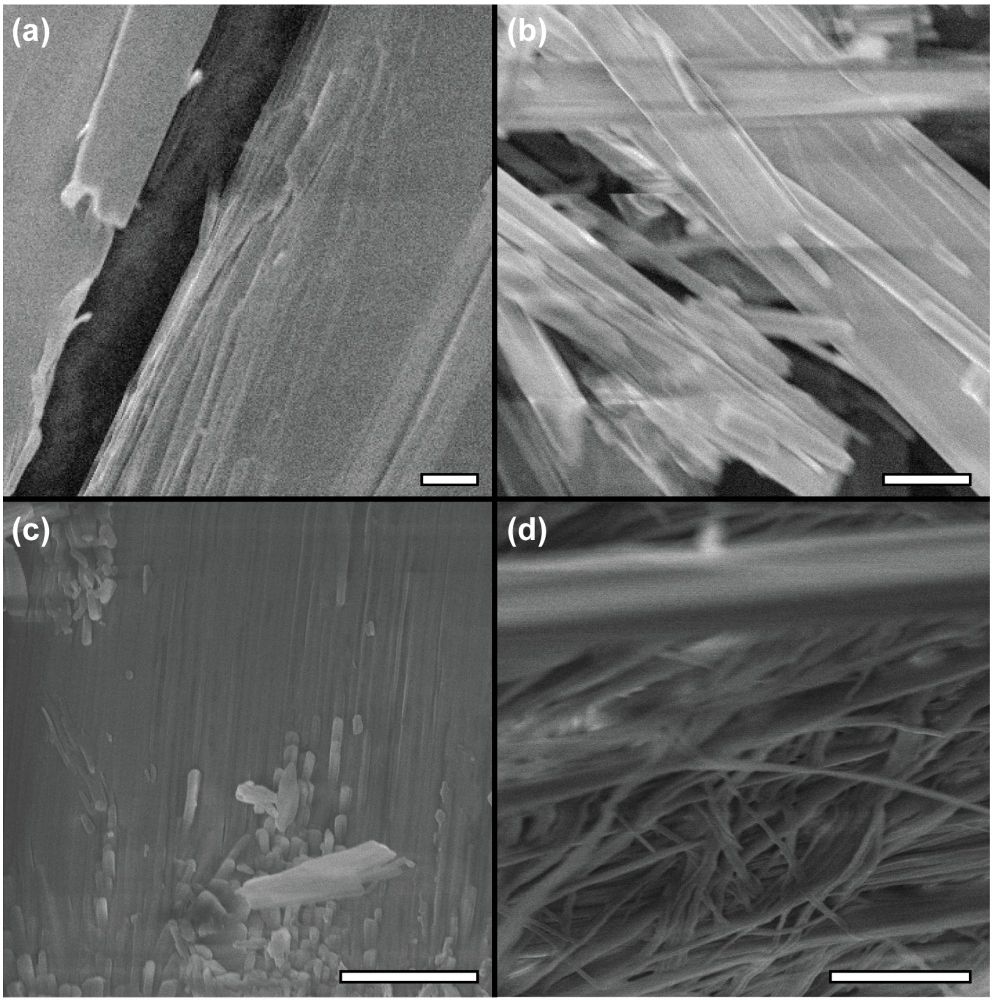

Figure S7. Xerogel SEM, all scale bars 5 µm. (a) Gelator **4** from cyclohexane, (b) gelator **6** from cyclohexane, (c) gelator **5** from cyclohexane (d) gelator **5** from petroleum ether.

Gelator Recovery

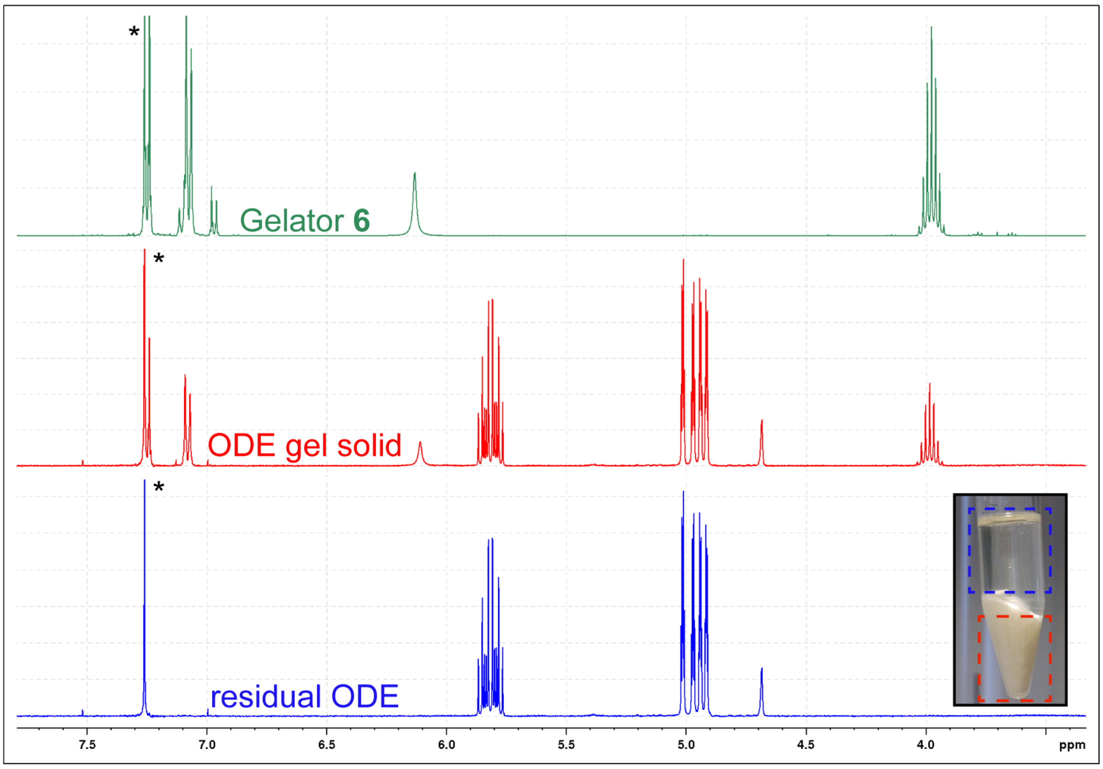

Figure S8. ^{1}H NMR demonstrating recovery of gelator and organic liquid by shearing and centrifugation of the gel. A 5 wt% gel of **6** in ODE was vigorously sheared and then centrifuged, resulting in pure ODE in the top layer and a crude gelator mixture at the bottom. Some liquid phase remained in the sample due to sampling methods. The inset photo shows the centrifuged sample with the boxes illustrating the sampling regions for the relevant spectra.
